# Amylopectin-*g*-Poly(Acrylic Acid): Synthesis and Application as Reduction Agent for In Situ Formation of Gold Nanoparticles

**DOI:** 10.3390/polym18131636

**Published:** 2026-07-01

**Authors:** Melinda-Maria Bazarghideanu, Marius-Mihai Zaharia, Florin Bucatariu, Ana-Lavinia Vasiliu, Marcela Mihai, Stergios Pispas

**Affiliations:** Petru Poni Institute of Macromolecular Chemistry, Romanian Academy, 41A Grigore Ghica Voda Alley, 700487 Iasi, Romania; melinda.bazarghideanu@icmpp.ro (M.-M.B.); zaharia.marius@icmpp.ro (M.-M.Z.); fbucatariu@icmpp.ro (F.B.); vasiliu.lavinia@icmpp.ro (A.-L.V.)

**Keywords:** amylopectin, pH-responsive polymers, copolymer, gold nanoparticles

## Abstract

A biological/synthetic hybrid graft copolymer was obtained by grafting poly(acrylic acid) (PAA, synthesized via reversible addition-fragmentation chain transfer (RAFT) polymerization) to amylopectin (AMP). The novel graft copolymer presents amphiphilic properties due to the inherent insolubility of AMP in water and was further utilized as a mediator for the synthesis of gold nanoparticles (AuNPs) following an environmentally friendly in situ procedure. The AMP-*g*-PAA copolymer formation by the interaction of the PAA end groups with the C(6)-OH groups on an AMP backbone was confirmed by Attenuated Total Reflectance-Fourier Transform Infrared (ATR-FTIR) and 1D (proton (^1^H NMR) and carbon (^13^C NMR) nuclear magnetic resonance, and Distortionless Enhancement by Polarization Transfer (DEPT)) and 2D (correlation (COSY) and heteronuclear single quantum coherence (HSQC)) spectroscopies. The calculated degree of substitution of 1.17 suggests that the grafting was done at one OH from the three in an anhydroglycosidic unit (AGU) (preferably at that in C6 position), with a mean grafting efficiency of 76%. Additional information obtained using thermogravimetric analysis shows that the thermal decomposition of AMP-*g*-PAA occurs in two steps, with a residual mass of ~16 wt% at 700 °C, higher than AMP or PAA, indicating increased thermal stability of the copolymer. Dynamic and electrophoretic light scattering (DLS and ELS) measurements were used to determine the hydrodynamic size and ionic charge of the AMP-*g*-PAA self-assemblies in aqueous solution as well as their stability. The AMP-*g*-PAA was subsequently tested as a reducing agent in the environmentally friendly synthesis of AuNPs in aqueous solution, at different incubation temperatures, reaction duration, and inorganic/polymer weight ratios. The development of the surface plasmon resonance band of AuNPs, observed in UV–vis spectra, was consistently monitored over the reaction time. DLS analysis indicated time-dependent changes in the AuNPs’ particle size distributions, while scanning transmission electron microscopy confirmed that the AuNPs formed at the inorganic/polymer weight ratio of 0.36 and at 60 °C were predominantly well-dispersed, spherical-shaped nanoparticles. The AuNPs synthesized in situ within the copolymer matrix did not introduce additional cytotoxicity compared to the parent copolymer alone, with the composites representing a promising safety baseline for further investigation in biomedical applications.

## 1. Introduction

The increasing requirement for sustainable materials and eco-friendly chemical methods has driven studies into polymeric systems from renewable sources [1,2,3]. Among the most promising types of renewable polymers are polysaccharides, as most of them are abundant, biodegradable, and biocompatible and have a high level of structural variability [4]. The reactive functional groups on their molecular structures offer a tremendous number of possibilities for chemical modification, which can facilitate the creation of new functionalized materials designed for biotechnology, drug delivery, nanotechnology, and many more applications [4,5,6]. Despite their versatility, polysaccharides have some disadvantages, especially in terms of mechanical properties and stability at high temperatures [7], which can be improved by their modification with synthetic polymers, thus developing hybrid materials with synergetic characteristics [8,9,10,11]. One example of polysaccharide is amylopectin (AMP), the branched constituent of starch, connected via α-(1,4) and α-(1,6) glycosidic bonds [12]. Depending on the starch source (wheat, rice, potato, etc.), AMP has different characteristics and properties (i.e., chain length, molecular mass, gelatinization temperature, crystallinity, solubility, etc.) [13], which in turn influence its applications in the food industry [14,15], cosmetics [16,17], or medicine [18,19]. Due to a large number of free –OH groups, AMP is able to undergo chemical modifications by various methods, i.e., by esterification, etherification, cross-linking, and grafting [20], yielding AMP derivatives with improved properties and extended applications. Grafting is a common chemical method for introducing new functional groups on the polysaccharide backbones and is considered one of the easy techniques that does not affect their inherent properties [21,22]. The “grafting from” method, where the polymerization of monomers takes place directly on the polysaccharide backbone through active sites generated by an initiator, has been the most widely used approach for the generation of polysaccharide derivatives. In contrast, the “grafting to” method, which involves attaching previously obtained synthetic polymers to the polysaccharide backbone via a coupling reaction, is less commonly used although it has gained increased attention in recent years [21,23,24]. The use of pre-synthesized polymers with suitable terminal functional groups allows excellent control and reproducibility of the copolymer molecular structure. Moreover, the method could potentially enable the design of more complex/versatile structures by grafting block copolymers or stimuli-responsive polymers to the polysaccharide, leading to new properties and advanced applications of the obtained materials. Over the last few years, the use of reversible addition-fragmentation chain transfer (RAFT) polymerization has become a more and more popular tool for performing controlled/living radical polymerizations of monomers [25]. Due to its controlled ability to produce polymers with well-defined architectures and narrow molecular weight distribution with tailored end group functionalities, RAFT is an ideal method for synthesizing functional polymers such as vinyl polymers. In particular, the attachment of vinyl polymers to polysaccharides has been investigated to enhance properties including but not limited to solubility, thermal stability, mechanical strength, and environment responsive capabilities [26,27]. Poly(acrylic acid) (PAA) is a flexible and ionizable weak polyelectrolyte that has been used in obtaining graft copolymers due to its hydrophilicity, pH responsiveness, biocompatibility, and ability to chelate certain metal ions [27,28]. Several studies are reported in the literature dealing with the grafting of PAA onto different polysaccharides, such as carboxymethyl cellulose [29], sodium alginate [30,31], chitosan [32,33], starch [34], amylose [35], etc. In the case of amylopectin, the literature reports only a limited number of studies on the “grafting from” method for the synthesis of PAA grafted derivatives [36,37], whereas no studies employing the “grafting to” approach have been reported, with this area remaining unexplored. In this context, the use of a PAA homopolymer pre-synthesized by RAFT for grafting onto AMP offers significant advantages for the design of a new AMP-*g*-PAA copolymer with enhanced control over molecular structure and reproducibility.

Over the past few years, polysaccharides and some of their derivatives have gained increasing interest in the production of the metal nanoparticles, serving both as reducers of the metallic ions as well as stabilizing agents for the resulting nanoparticles through mechanisms of steric and electrostatic repulsion [38,39,40]. Almost all the already-known conventional nanoparticle synthesis methods, although efficient, utilize toxic reagents to reduce the metallic ions or use organic solvents in the synthesis process, consuming large amounts of energy for synthesis, all of which are not compatible with green chemistry principles [8,41]. Therefore, due to the growing concern regarding the environmental and health impacts of conventional nanoparticle synthesis processes, the green synthesis of metal nanoparticles has arisen as an important area of research. Polysaccharide-assisted methods of synthesizing nanoparticles provide an environmentally friendly means for producing metal nanoparticles, enhancing their colloidal stability and biocompatibility, and often reducing the production cost while offering fewer steps in the synthesis process [8,41,42].

Gold nanoparticles (AuNPs) are particularly acknowledged for their distinct optical, electronic, and catalytic characteristics and have received considerable attention due to their chemical stability as well as their potential to serve through the localized surface plasmon resonance (LSPR) phenomenon in the application of biosensors, biomedical imaging, drug delivery, and catalysis [43,44,45]. The LSPR phenomenon occurs through free electron oscillation at the nanoparticles’ surface as a consequence of exposure to light and is visually distinguished by the appearance of a specific coloration depending on the size and shape of the gold nanoparticles. As such, these parameters highly influence AuNPs properties and subsequently their applicability. Even more, due to their high surface energy, AuNPs have a tendency to aggregate, hence the need to introduce stabilizing agents in the system. Polysaccharides possess numerous reactive functional groups on their molecular structure that act as stabilizers or reducing agents (–COOH, –NH_2_, –OSO_3_H, –CHO, and –OH). Ideally, in order to eliminate specific reducing agents from the reaction, such as citrates, the chosen polysaccharide should contain functional groups able to reduce [AuCl_4_]^−^ in situ, depending on the reaction conditions (temperature, pH, reaction time, etc.). AMP has a high content of hydroxyl groups, which can act as both reducing agents as well as active sites for potentially grafting synthetic polymers. Thus, AMP can provide a sustainable and biodegradable polymeric network and, when grafted with synthetic polymeric chains, exhibits enhanced ability to bind metal ions, better reduction efficiency, and improved confinement of nanoparticles on the nanocomposite surface [7]. The literature shows that poly(acrylic acid) (PAA) has carboxyl groups that can deprotonate at pH values above pKa (pKa = 4.5) [46], hence its ability to interact with Au^3+^ ions to facilitate the reduction to Au^0^ [47,48].

Although there have been some notable advances in the synthesis of polysaccharide copolymers obtained by the “grafting to” method and on the green processes for synthesizing inorganic nanoparticles (e.g., bio-based or bio-derived), according to our knowledge, no studies have been presented up to now on the synthesis of AMP-*g*-PAA copolymer and its capability to act as reducing agent for gold ions, resulting in composites with AuNPs. As such, in the current study, RAFT polymerization was applied to obtain the PAA homopolymer, which was subsequently grafted directly onto the AMP main chain. The characterization of the resulting grafted copolymer was performed by utilizing Attenuated Total Reflectance-Fourier Transform Infrared (ATR-FTIR) and 1D (proton (^1^H NMR) and carbon (^13^C NMR) nuclear magnetic resonance, and Distortionless Enhancement by Polarization Transfer (DEPT)) and 2D (correlation (COSY) and heteronuclear single quantum coherence (HSQC)) spectroscopies, thermogravimetric analysis (TGA), and dynamic and electrophoretic light scattering (DLS and ELS) measurements. Afterwards, AuNPs were obtained by using AMP-*g*-PAA, which acts both as a reducing and stabilizing component in the formed composites. The formation of composites with AuNPs were intensely investigated under various reaction conditions (ratio between components, temperature, and incubation time), and the copolymer/AuNPs system presenting the best physicochemical results was evaluated in terms of its cytotoxicity.

## 2. Materials and Methods

### 2.1. Materials

Amylopectin from maize (AMP), acrylic acid (AA), 2,2′-azobis(isobutyronitrile) (AIBN), 1,4-dioxane, n-hexane, potassium persulfate (KPS, 99%), 4-cyano-4-[(dodecylsulfanylthiocarbonyl)sulfanyl] pentanoic acid (CDSPA), and tetrachloroauric(III) acid solution (HAuCl_4_, 99.99% trace metals basis, 30 wt% in dilute HCl) were purchased from Sigma-Aldrich (Sigma Chemical Co.; St. Louis, MO, USA) and were used as received. Other materials used included dimethyl sulfoxide (DMSO) from Lach-Ner, Ltd., Tovární, Neratovice, Czech Republic, and hydrochloric acid (HCl), sodium hydroxide (NaOH), and ethanol (EtOH) from Chemical Company SA, Iasi, Romania. All aqueous solutions were prepared in ultrapure water (conductivity 0.552 μS/cm) clarified by an EVOQUA Ultra Clear TPTWF Systems (Evoqua Water Technologies LLC; Barsbüttel, Germany).

### 2.2. Grafting of Poly(Acrylic Acid) Homopolymer Synthesized by RAFT Polymerization to Amylopectin

**Scheme 1:**
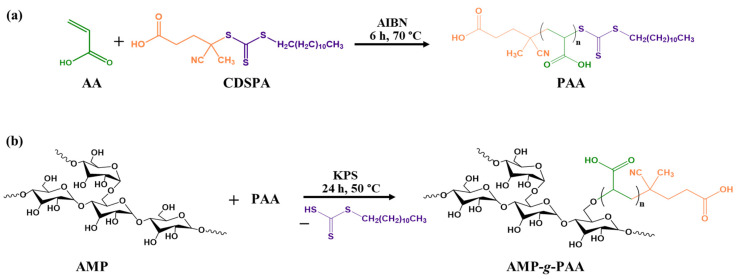
(**a**) RAFT polymerization of acrylic acid and (**b**) synthesis of AMP-*g*-PAA.

The AMP-*g*-PAA copolymer was synthesized as described in Scheme 1, adapting previously described synthesis protocols [33,49,50].

Thus, the first step involves the polymerization of AA by applying a RAFT polymerization procedure (Scheme 1a), providing well-defined control over both the degree of polymerization and the end group functionalities of the resulting polymer. Herein, the AA (6.9 × 10^−2^ moles) polymerization was carried out in the presence of the radical initiator AIBN (5.6 × 10^−5^ moles) and CDSPA as chain transfer agent (CTA) (5.6 × 10^−4^ moles) at a molar ratio of [AIBN]: [CDSPA] = 1:10, with 1,4-dioxane as solvent (25 mL). The synthesis took place in an inert nitrogen atmosphere at 70 °C for 6 h. After the reaction time was reached, PAA was precipitated in n-hexane (250 mL) for purification and separation from the reaction environment, followed by drying under vacuum for 5 days. The size-exclusion chromatography (SEC)-yielded information for the obtained PAA is as follows: Mn = 9400 g·mol^−1^, Mw = 11,500 g·mol^−1^, and Ð = 1.22 (Figure S1), and the determined polymerization yield was 93.6%. The obtained PAA retains at one chain-end the thiocarbonylthio group that can subsequently interact with the -OH groups on AMP. In the second step (Scheme 1b), the AMP-*g*-PAA copolymer was obtained by a “grafting to” approach, namely by attaching the obtained PAA to the AMP backbone via a radical mediated coupling reaction using KPS as the initiator for the grafting reaction. In the reaction conditions, KPS forms sulphate radicals, which can attack both the polysaccharide -OH groups (extracting the hydrogen atom and forming macroradicals) and the PAA end groups (leading to radical-induced cleavage of the thiocarbonylthio end group and forming the PAA macroradical). The further interaction between the formed macroradicals facilitate the grafting of PAA to AMP chains. Practically, 0.5 g AMP fully dissolved in DMSO (30 mL, 30 min, room temperature) was mixed in a round-bottom flask with 5 mL aqueous solutions of PAA (0.3 g) and 1 mL KPS (0.0064 g). The grafting reaction was performed under inert atmosphere (nitrogen degassing for 15 min) at 50 °C for 24 h under continuous stirring. Finally, the copolymer was dialyzed against ultrapure water for 5 days at room temperature and dried by lyophilization using an ALPHA 1–2LD plus lyophilizer (Donau Lab., Kiev, Ukraine). The grafting efficiency (GE) of PAA onto AMP of approximately 76% was calculated using Equation (1) [51]:

(1) $$GE\left(\%\right)=\frac{W_{AMP-g-PAA}}{W_{AMP}+W_{PAA}}\times 100$$

where *W*_AMP*-g*-PAA_, *W*_AMP_, and *W*_PAA_ are the dry weights of AMP-*g*-PAA, AMP, and PAA, respectively.

### 2.3. AMP-g-PAA/AuNPs Composite Preparation

The preparation of the AMP-*g*-PAA/AuNPs composite involved the use of HAuCl_4_ We agree as a precursor for AuNPs, with the AMP-*g*-PAA copolymer being used as a reducing and stabilizing agent. Thus, the in situ reduction of HAuCl_4_ (4.19 mM) was performed in AMP-*g*-PAA aqueous solution (0.10 mg·mL^−1^, pH = 4.8) at two different weight ratios: R = HAuCl_4_/AMP-*g*-PAA = 0.28 (mixture pH of 4.0) and 0.36 (mixture pH of 3.7), which were controlled by the HAuCl_4_ volume used in each experiment (100 and 125 µL, respectively), while the volume of the AMP-*g*-PAA aqueous solution was maintained constant at 5 mL. The formed mixtures were first heated at 40 °C, 50 °C, or 60 °C in a thermostatic water bath for different periods of time (1–6 h). After that, the resulting samples of copolymer/AuNPs were kept at room temperature (25 °C) for 10 days and characterized daily by UV–vis spectroscopy and DLS.

### 2.4. Characterization Methods

The number and weight-average molecular weight (Mn and Mw) and dispersity (Đ) of PAA were determined by performing size-exclusion chromatography (SEC) analysis. The SEC measurements were carried out using an OMNISEC multidetector GPC/SEC system (Malvern Pananalytical Limited, Malvern, Worcestershire, UK) equipped with a PL-EMD 950 evaporative mass detector (Polymer Laboratories, Long Beach, CA, USA) using a PL aquagel OH 40 Agilent column. The temperature inside the columns was set at 40 °C, and an H_2_O + 0.02% NaN_3_ solution was used as the solvent at a flow rate of 1 mL·min^−1^. The calibration measurements were performed using polyethylene oxide standards.

The ATR-FTIR spectra were obtained using the IR Tracer-100 spectrometer (Shimadzu Corporation, Kyoto, Japan), with a GladeATR module (PIKE Technologies, Madison, WI, USA), in the range 400 cm^−1^ to 4000 cm^−1^ and a resolution of 4 cm^−1^ at 25 °C. Each spectrum consisted of an average of 256 scans. The ACD/Spectrus Processor software (version 2023.2.4; Advanced Chemistry Development Inc., Toronto, ON, Canada) was utilized to conduct spectral processing and interpret the spectral data. For comparison, a physical mixture of AMP and PAA having the same weight ratio as that used in the copolymer synthesis was prepared, and its spectrum was registered.

The ^1^H and ^13^C NMR spectra of the AMP, PAA, and AMP-*g*-PAA polymers were recorded using a Bruker Neo-1 400 MHz NMR spectrometer (Bruker, Rheinsteitten, Germany) equipped with a 5 mm QNP direct detection probe and z-gradients at 25 °C. Each polymer sample was dissolved in 800 µL DMSO-d6 and held at 25 °C under continuous stirring for 10 min to fully dissolve. The Bruker TopSpin Spectrus Processor software (version Bruker TopSpin 4.3.0 (2023); Bruker, Rheinsteitten, Germany) was utilized to conduct spectral processing and interpret the spectral data. Peaks located near 2.5 ppm for ^1^H NMR and 39.5 for ^13^C NMR represent characteristic peaks from the DMSO-d6 solvent, while an extremely high peak (3.4 ppm) in ^1^H NMR spectra represent hydrogen from the water in the solvent. Also, ^13^C NMR DEPT was used to determine the number of hydrogen atoms attached to each carbon atom in a molecule, with a pulse angle of 135°, where -CH_3_ and -CH- carbons appear upright (positive peaks), >CH_2_ carbons appear inverted (negative peaks below the baseline) whereas quaternary carbons (carbons with no attached hydrogens), or -OH groups do not appear in any DEPT spectrum.

The degree of substitution (DOS) in the obtained copolymer was determined using the ^1^H NMR spectrum of AMP-*g*-PAA from the ratio of the integral at 2.22 ppm (attributed to the three protons in positions a and d in PAA) to that at 5.1 ppm (assigned to the anomeric proton of AMP, position 1), according to Equation (2) [50], as follows:

(2) $$DOS=\left(\frac{{3\times I}_{2.2}}{I_{5.1}}\right):3$$

Two-dimensional NMR spectra, such as H,H correlation spectroscopy (COSY) and H,C heteronuclear single quantum coherence (HSQC), were recorded using standard pulse sequences as delivered by Bruker with TopSpin 4.0.8 spectrometer control and processing software.

The thermal behavior of AMP, PAA, and AMP-*g*-PAA was analyzed by using a thermogravimetric analyzer type Discovery TGA 5500 (TA Instruments, New Castle, DE, USA). Each sample of approximately 6 mg was placed in a platinum plate and investigated under nitrogen atmosphere (40 mL·min^−1^) at temperatures between 37 and 700 °C, with a 10 °C·min^−1^ heating rate.

Dynamic and electrophoretic light scattering (DLS/ELS) measurements were performed by operating the Litesizer DLS 500 (Anton Paar, Graz, Austria) instrument utilizing a 40 mW semiconductor laser diode operating at 658 nm. The ELS measurements carried out for the synthetic homopolymer and the graft copolymer were performed at a scattering angle of 15° and at 25 °C; the reported zeta potential values were an average of 100 individual runs. The zeta potential measurements were performed first in ultra-pure water, followed by measurements in solutions of different pHs (between 3 and 10) adjusted by additions of 0.1 M HCl or 0.1 M NaOH aqueous solutions. The DLS measurements were conducted at a scattering angle of 90° and at room temperature after an equilibration time of 60 s for each experiment, representing a mean of 5 consecutive runs of 10 s duration. The size of the self-assembled structures formed by the AMP-*g*-PAA copolymer in aqueous solutions was also investigated both as a function of pH (in the pH range 3–10) adjusted by additions of 0.1 M HCl or 0.1 M NaOH aqueous solutions, respectively, and as a function of ionic strength, varied by addition of 1N NaCl aqueous solution (50–1000 µL). Additionally, the DLS measurements were performed to investigate the formation of AMP-*g*-PAA/AuNPs composites in the dispersed/solution phase in relation to the initial components’ weight ratio and reaction temperatures over a period of 10 days.

The UV–vis spectra were registered using a SPECORD 200 PLUS UV-Vis Spectrophotometer (Analytik Jena, Jena, Germany) to study the evolution of LSPR peaks associated with the formation of AuNPs. Quartz cuvettes with a 1 cm light path were used to measure each sample, and a baseline correction was performed using distilled water, the solvent used to prepare the composite solution.

The morphological characterization of the AMP-*g*-PAA/AuNPs composites was performed utilizing a Verios G4 UC Scanning Electron Microscope (Thermo Scientific, Brno, Czech Republic) with a detector for scanning transmission electron microscopy (STEM). The samples were deposited onto 300 mesh carbon film-coated copper grids, and the images were obtained for each sample using different magnifications. The average diameters of gold nanoparticles were estimated by measuring approximately 60–70 particles from each sample and using ImageJ software (version 1.52a) to calculate mean values and histograms representations.

The cytotoxicity of both the AMP-*g*-PAA copolymer and the AMP-*g*-PAA/AuNPs composites was evaluated using a Human dermal fibroblasts (HDF) cell line from Innoprot (Derio, Spain). Cells were seeded at a density of 10,000 cells per well in white opaque 96-well plates and allowed to adhere overnight under standard culture conditions (37 °C, 5% CO_2_, humidified atmosphere) prior to treatment in Alpha MEM Eagle (aMEM) with 10% fetal bovine serum (FBS) from PAN Biotech (Aidenbach, Germany) and 1% antibiotic–antimycotic solution from Capricorn Scientific (Ebsdorfergrund, Germany). Cell cultures were left overnight in the incubator to facilitate cell adhesion. The next day, different aliquots of copolymer/composite (0.125, 0.25, 0.5, and 1 µg·mL^−1^) were introduced over the cell wells, and the cell plates were incubated for another 48 h. After this time, the cell viability was assessed using the CellTiter-Glo^®^ Luminescent Cell Viability Assay (Promega, Madison, WI, USA), which quantifies intracellular ATP, a marker of metabolically active cells. Luminescence was recorded using a FLUOstar^®^ Omega microplate reader (BMG Labtech, Ortenberg, Germany). Relative cell viability (RCV) was expressed as a percentage relative to the viability of the control sample using Equation (3):

(3) $$RCV=100\times \frac{A_{s}-A_{c}}{A_{C}{-A}_{B}}$$

where *A_S_*, *A_B_*, and *A_C_* are the absorbances of the sample, of the blank, and of the control, respectively. Data are presented as mean ± standard deviation of at least three independent experiments, each performed in duplicate.

Statistical analysis was carried out using GraphPad Prism v.10.6.1 (GraphPad Software, Boston, MA, USA). The normality of data distribution was first confirmed using the Shapiro–Wilk test. Following confirmation of normality, differences in relative cell viability between treated groups and the untreated negative control were assessed by two-way analysis of variance (ANOVA) followed by Dunnett’s multiple comparisons test. A *p*-value < 0.05 was considered statistically significant.

## 3. Results

### 3.1. Characterization of AMP-g-PAA Copolymer

**Figure 1:**
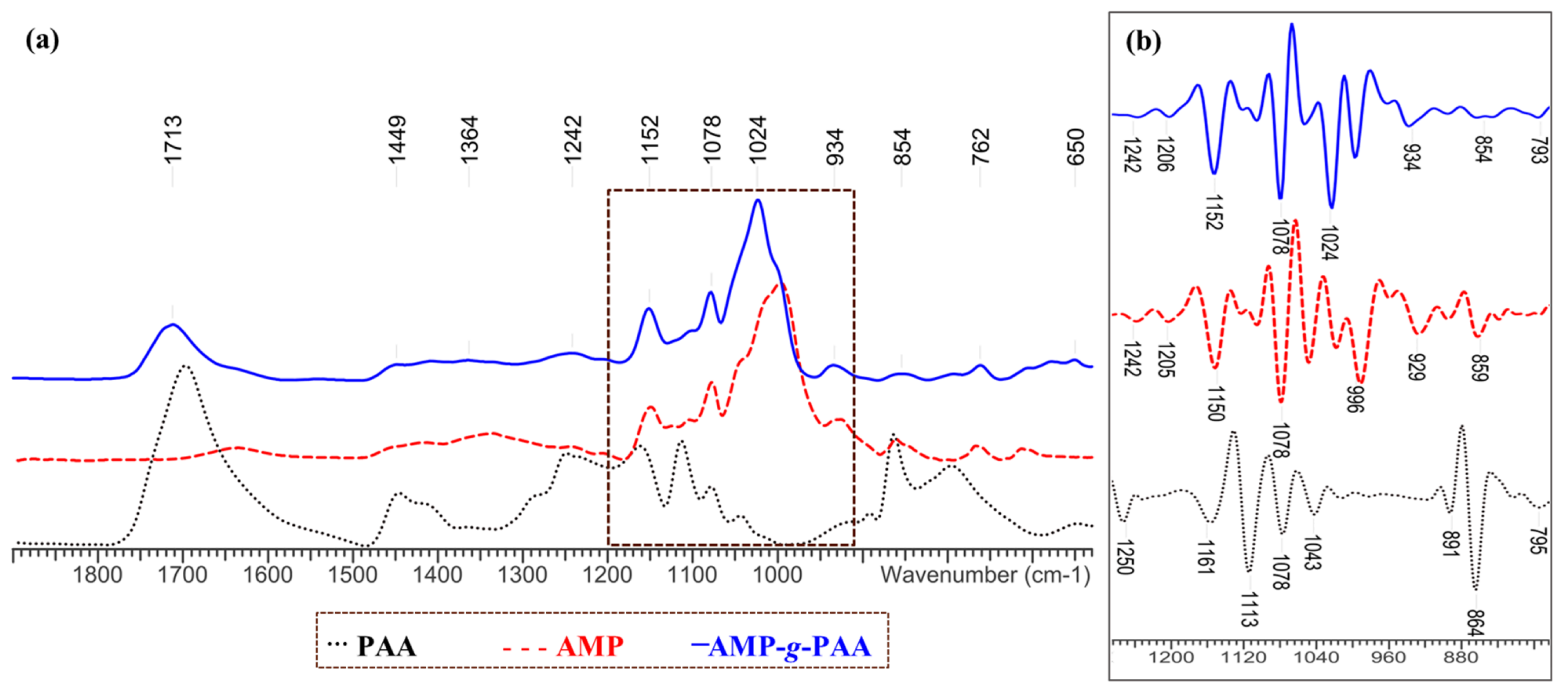
ATR-FTIR spectra of PAA, AMP, and AMP-*g*-PAA: (**a**) fingerprint area and (**b**) the second derivative of the selected area.

**Figure 2:**
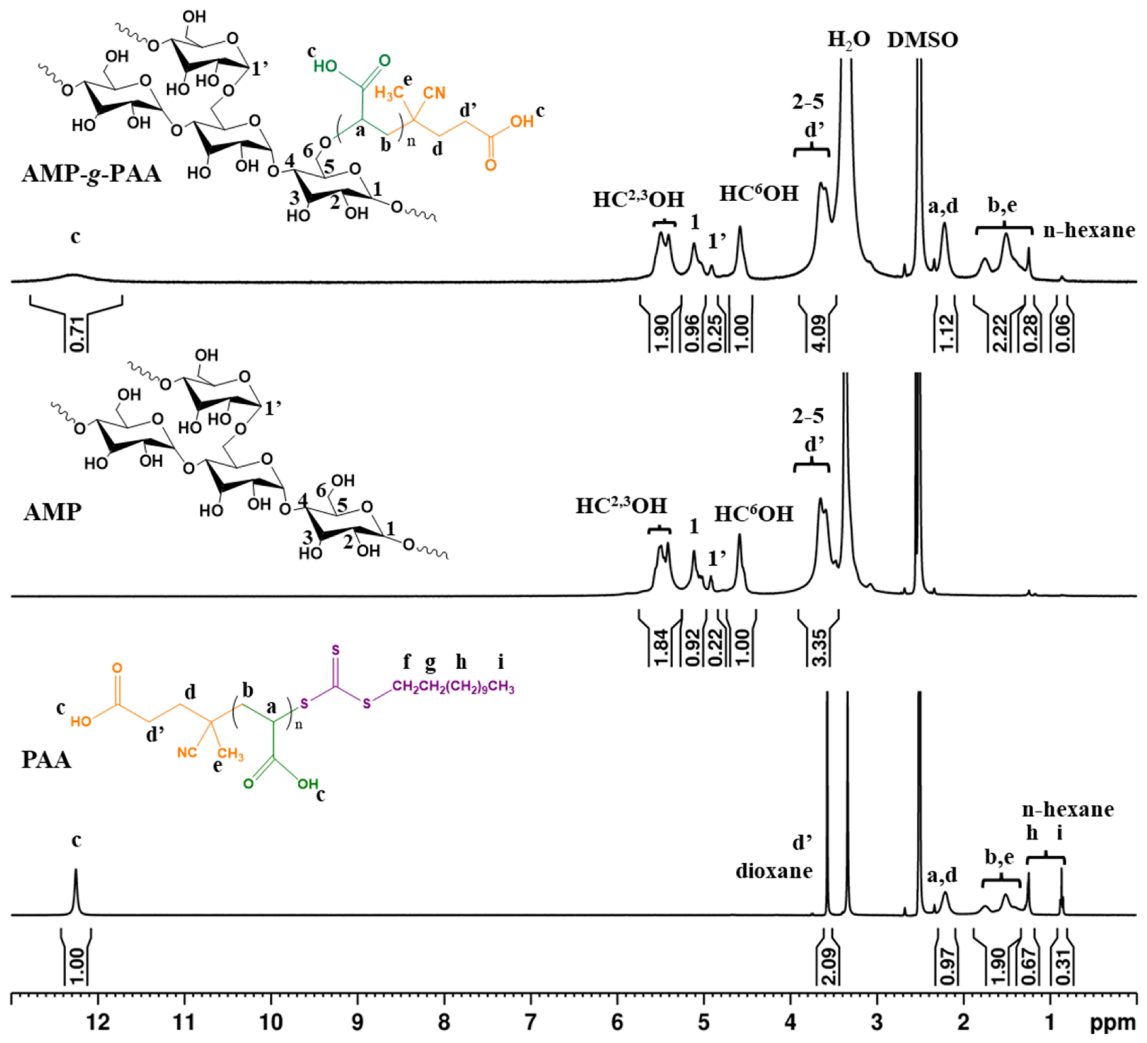
^1^H NMR spectra of PAA, AMP, and AMP-*g*-PAA.

The ATR-FTIR and ^1^H NMR analyses were performed aiming for the confirmation of the AMP-*g*-PAA copolymer chemical structure in comparison to the corresponding individual spectra of PAA and AMP (Figure 1 and Figure 2). Additionally, in order to further demonstrate the grafting of PAA onto the AMP backbone, the FTIR spectrum of the AMP-*g*-PAA copolymer was compared with those of AMP and a physical AMP/PAA mixture (Figure S2).

As seen in Figure 1, the FTIR spectrum of AMP-*g*-PAA contains both the characteristic absorption peaks of the AMP backbone and of the PAA side chains. More precisely, the FTIR spectrum of PAA displays an intense absorption peak at ~1700 cm^−1^ characteristic of the C=O bond asymmetric stretching of the carboxylic functional group, a signal that can be seen in the copolymer spectrum with a lower intensity at 1713 cm^−1^. The bands attributed to the stretching vibrations of CH and CH_2_ bonds of PAA can also be found in the 1460–1360 cm^−1^ region. The signals at 1152, 1078, and 1024 cm^−1^ are characteristic of the polysaccharide backbone and correspond to the C–O stretching vibrations, being associated with the C–O–C ether linkage and C–OH groups of the AGU rings, respectively. Also, the absorption peak at 1242 cm^−1^ is typically related to the stretching vibrations of the CH_2_–OH side chain of AMP and to the in-plane deformation of the PAA carboxylic OH group. The ATR-FTIR spectra (Figure 1a) and their corresponding second derivative (Figure 1b) highlight the shift of the stretching vibrations of the C–O bonds in AMP spectrum from 996 cm^−1^ to 1024 cm^−1^ in the AMP-*g*-PAA spectrum, suggesting the reaction of the –OH polysaccharide groups with PAA and the increase in the aliphatic ether bond content by the formation of new C–O–C ether linkage. This shift was not observed when the AMP/PAA physical mixture was analyzed (Figure S2), with the corresponding peak being found at 996 cm^−1^, similar to the AMP spectrum, further confirming the success of the grafting procedure. Additionally, the decrease in the signal intensity at 854 cm^−1^, corresponding to the C–OH bond in AMP, as well as the absence of the signals at 1113 cm^−1^ and 864 cm^−1^, corresponding to the C=S and C–S bond in PAA from the CTA, sustain the fragmentation of the chain end of PAA and its grafting onto the polysaccharide backbone. These spectral changes provide clear qualitative evidence of successful graft copolymer formation. However, due to the surface-sensitive nature of ATR-FTIR and band overlap in the fingerprint region, this technique does not allow for accurate quantitative determination of the grafting yield.

**Figure 3:**
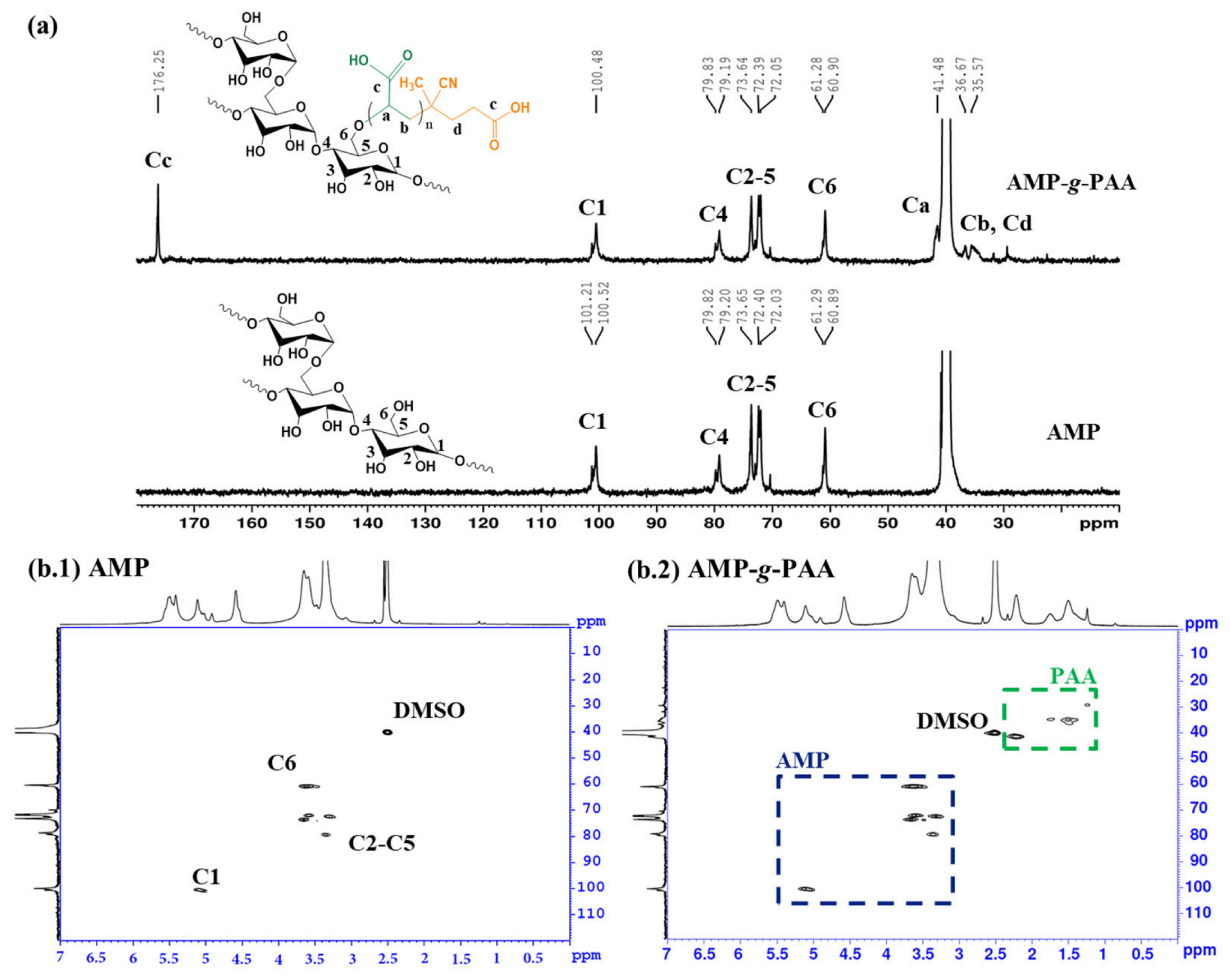
^13^C NMR (**a**) and HSQC (**b.1**,**b.2**) spectra of the copolymer AMP and AMP-*g*-PAA.

The structure of the graft copolymer was also validated using 1D (^1^H NMR, ^13^C NMR, and DEPT) and 2D (COSY and HSQC) spectroscopy (Figure 2, Figure 3 and Figure S3). The ^1^H NMR (Figure 2) spectrum of AMP showed the expected carbohydrate resonances, including the peaks around 4.9–5.1 ppm, which correspond to the α-(1,4) and α-(1,6) anomeric protons (positions 1 and 1′); a broad peak between 3.3–3.7 ppm, associated with the protons from the anhydroglucose units (positions 2–5); and the sharp peaks near 4.6 ppm and 5.4–5.5 ppm, which indicate the hydroxyl group protons (positions 6 and 2, 3, respectively). The ^1^H NMR spectrum of PAA shows a very sharp peak around 12.2 ppm (due to carboxylic OH) and strong signals ascribed to the aliphatic part of the polymer with peaks located at 2.2 ppm (backbone CH(a) and CH_2_(d) from CTA) and at 1.3–1.9 ppm (backbone CH_2_(b) and CH_3_(e) from CTA). Additionally, the signal corresponding to the CTA protons from the methyl groups in positions h and i are located at 0.89 ppm and 1.24 ppm, respectively, and overlap to n-hexane protons signals. Also, the CH_2_(d’) from the CTA signal at 3.6 ppm overlaps with that of CH_2_ from dioxane.

The AMP-*g*-PAA ^1^H NMR spectrum contains peaks both from the preserved AMP structure (the hydrogen peaks from –CH and –CH_2_ groups appeared at 3.5–3.9 ppm, and the anomeric hydrogen peaks appeared at 4.9–5.2 ppm), but also distinct signals between 1.3–2.3 ppm originating from the aliphatic structure of PAA and the chain end remaining from the CTA. As well, the broadened/attenuated peak near 12 ppm is indicative of the protons of the carboxylic group –COOH of PAA. Furthermore, the significant reduction in the signal at 0.89 ppm in the copolymer spectrum, originating from the methyl protons of the PAA chain ends (position i), which also overlap with the n-hexane protons, suggests the fragmentation of the synthetic polymer and its attachment onto AMP. The slight decrease in the ratio between the integrals of the protons of OH in position 6 and that of OH in positions 2 and 3 corresponds to the decrease in that in position 6, suggesting that the grafting reaction occurs most probably on the -OH groups at C6. This is reasonable considering the lower steric hindrance of the particular OH groups [52]. To calculate the DOS via ^1^H NMR, the integral values from the AMP-*g*-PAA spectrum were used, which correlate the signal at 2.2 ppm (three positions a and d in PAA) to the proton signal at 5.1 ppm (the anomeric proton from anhydroglucose units of AMP, α-(1,4) glycosidic bonds, position 1). Using Equation (2) from this paper along with the ^1^H NMR integral value data, a DOS of 1.17 was obtained, suggesting that the grafting reaction took place at one OH from the three in AGU (preferably at that in C6 position), with a mean grafting density of one PAA chain to three AGU units [50].

The AMP-*g*-PAA ^13^C NMR spectrum (Figure 3) also contains peaks assigned both to AMP (in the range 50–110 ppm) [53,54] and from PAA backbone (up to 45 ppm, some overlapping with the DMSO-d6) and the -COOH groups (at 176 ppm). The signals displayed at 60.43–60.82 ppm in both the AMP and AMP-*g*-PAA spectra are attributed to the carbons C6 likely involved in the grafting process. The reduction in intensity of the C6-associated signal in the AMP-*g*-PAA spectrum as compared to the similar C6 spectrum of APM indirectly (since the method does not detect the modification of -OH groups) suggests that a part of the -OH groups on C6 sites was grafted, and part of them remained unreacted. The DEPT spectra recorded at a pulse angle of 135° enables the differentiation of carbon atom types based on their protonation state, with the methylene carbons being identified by signals of negative phase (Figure S3a) since the quaternary carbons (non-protonated) can be detected only in the conventional ^13^C NMR spectrum (Figure 3a) [55].

As a widely used 2D NMR technique that maps which protons are coupled to each other through chemical bonds, the AMP-*g*-PAA COSY spectrum shows cross-peaks between neighboring protons, confirming their sequential connectivity along the AMP backbone (Figure S3(b.1)) as follows: H1 to H2, H2 to H3, and H3 to H4, with cross-peaks in the range 2.9–3.7 ppm/4.9–5.7 ppm [53,54,55]. Also, the correlation signals of the protons from PAA appeared in the copolymer 2D spectra, being visible a prominent cross-peak at (1.6 ppm/2.3 ppm), which represents the scalar coupling between the–CH_2_– and –CH– units within the continuous PAA graft chains and 0.85 ppm/1.2 ppm, which evidences the RAFT CTA end group. Also, the absence of the cross-peaks at about 4.6 ppm/1.9 ppm (assigned to -CH(S) end groups), confirms the absence of the trithiocarbonate end groups of a living RAFT agent in the copolymer structure. Some minor changes in the correlation signal at 4.6 ppm/3.5 ppm, assigned to hydroxyl group protons at C6, could also suggest some changes in the environmental interaction and their transformation in ether groups (which were not discriminated in H,H COSY spectra). Also, the AMP-*g*-PAA spectrum shows a unique cross-peak at 4.2 ppm, connecting it directly to the beginning of the aliphatic PAA signals (1.2 ppm), which serves as structural proof of a covalent attachment rather than a simple physical blend of the two polymers.

The heteronuclear two-dimensional HSQC was also used to evidence the correlations between protons and the carbon atoms (Figure 3(b.1,b.2)) [55]. Thus, both AMP and AMP-*g*-PAA HSQC spectra evidenced the polysaccharide backbone structure as C1 at 4.9–5.25/98–103 ppm, C2–C5 at 3.1–4.8/70–80 ppm, and C6 at 3.3–3.8/58–62 ppm. Also, the presence of PAA in the copolymer structure is evidenced by the correlations at 1.3–2.5/30–45 ppm assigned to methylene and methine groups on the synthetic PAA chain body. Taking into account that the OH in the -CH_2_-OH groups typically does not appear in these correlation spectra since the experiments detect direct correlations only between carbons and their directly attached protons (^1^H and ^13^C bonded via one bond), the method does not allow clear evidence of the grafting at these groups. Nevertheless, the absence of the HSQC correlation at 4.6–4.8/48/52 ppm, which is correlated to a specific carbon holding onto the thiocarbonyl group (living chain end), confirms once again the absence of the trithiocarbonate end groups of PAA and thus the proposed copolymer structure in Scheme 1.

**Figure 4:**
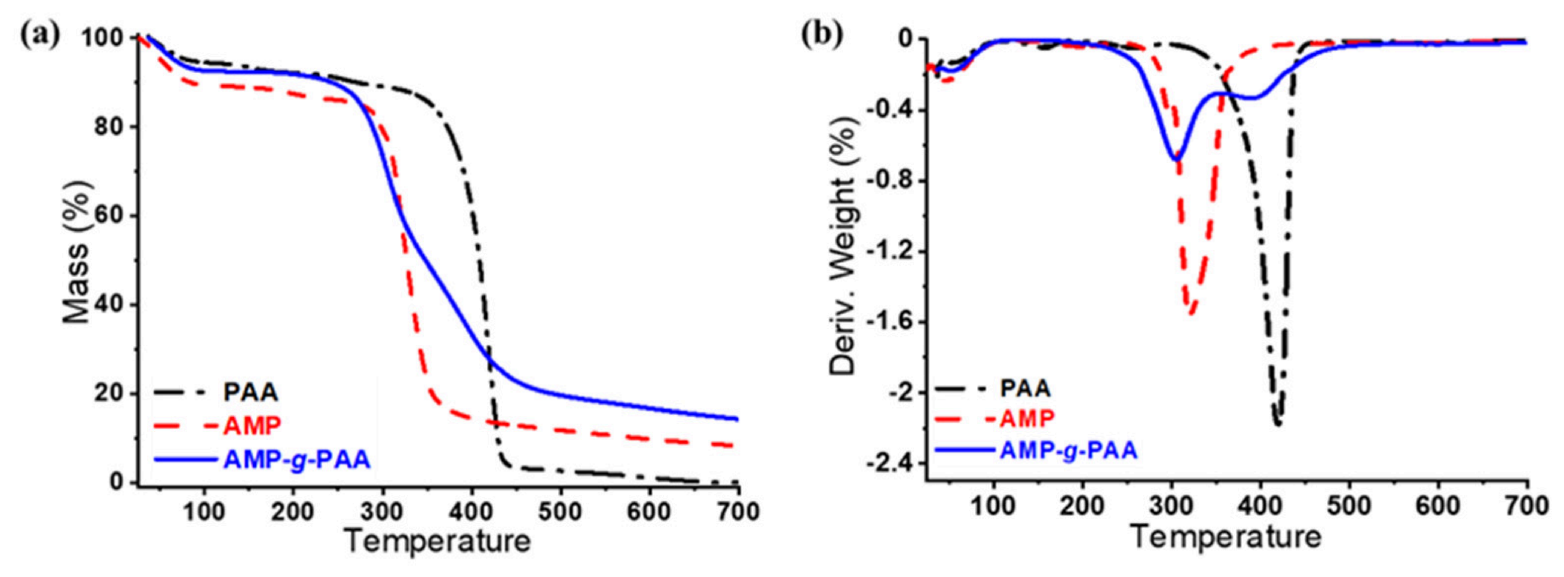
Thermogravimetric analysis (**a**) and the corresponding derivative (**b**) curves of the PAA, AMP, and AMP-*g*-PAA.

Thermogravimetric analysis was performed to evaluate the thermal stability of the synthetized AMP-*g*-PAA copolymer (Figure 4) in comparison with that of the starting components, AMP and PAA. According to the thermogravimetry curves, all analyzed samples presented an initial stage with a small weight loss at relatively low temperatures (~60 °C), which corresponds to the evaporation of the residual solvent and the absorbed water from the environment. AMP presents a significant thermal degradation step (~70% weight loss) in the 260–390 °C temperature range, which is associated with the decomposition of the polysaccharide backbone, resulting in a char residual mass of ~10 wt% at 700 °C.

In contrast, PAA shows a better thermal stability in the 100–390 °C range, with the complete thermal decomposition via the processes of decarboxylation and/or chain scission at ~420 °C leaving almost no residual mass [56]. By comparison, the thermal degradation profile for AMP-*g*-PAA changed from those of the initial constituents, reflecting the combination of thermal behavior from AMP and the PAA grafted onto it. Thus, a thermal degradation in two stages took place, with the first stage in the range 250–350 °C (~40% weight loss) induced mainly by decomposition of AMP and the second in the range 360–460 °C (~25% weight loss) specific to the degradation of the PAA part. The thermogravimetry curve for AMP-*g*-PAA shows a residual mass of ~16 wt%, higher than AMP (10 wt%) or PAA (almost 0), after reaching 700 °C, indicating increased thermal stability of the copolymer, most probably due to the occurring strong intermolecular interactions between the AMP main chain and PAA grafted side chains.

In conclusion, the above-presented results obtained by ^1^H NMR and ATR-FTIR spectroscopies and TGA/DTG analysis provides complementary structural and quantitative evidence for the formation of the AMP-*g*-PAA graft copolymer.

### 3.2. AMP-g-PAA Behavior in Aqueous Solution

**Figure 5:**
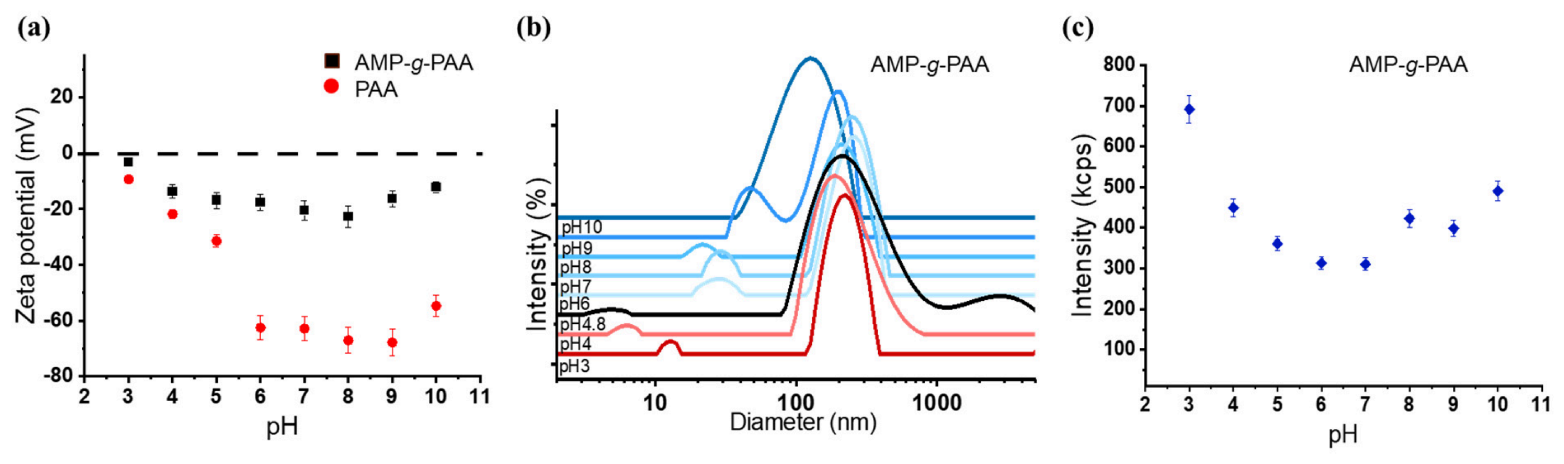
The pH influence on the (**a**) PAA and AMP-*g*-PAA zeta potential values and on the (**b**) size distribution and (**c**) scattered intensity of self-assembled AMP-*g*-PAA structures in aqueous solution.

**Figure 6:**
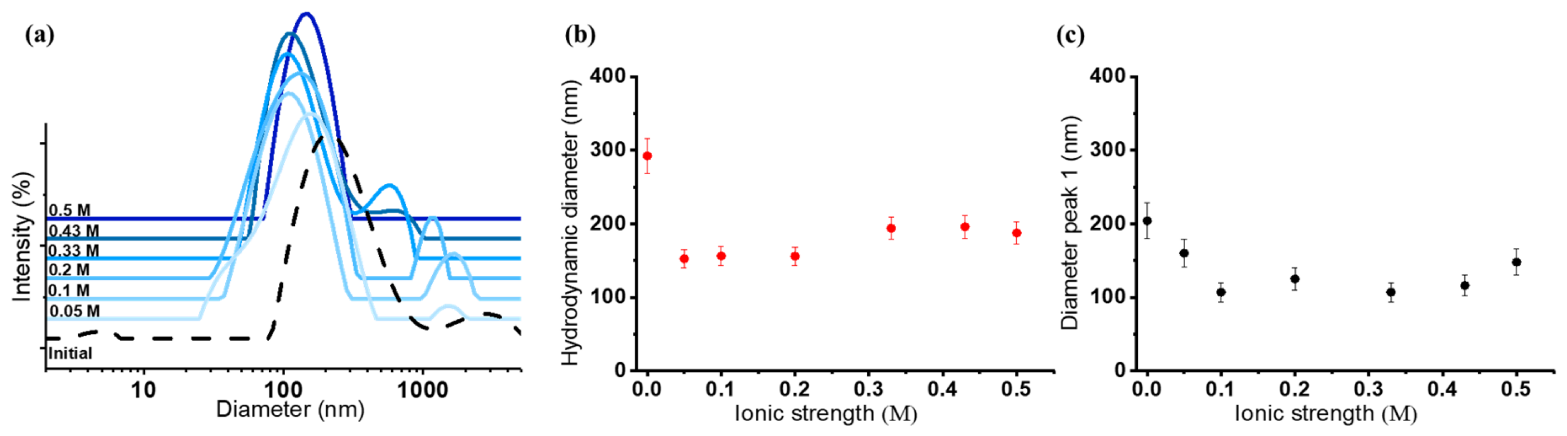
Size distribution: (**a**) hydrodynamic diameter (**b**) and mean diameter resulted from the major peak size distribution (**c**) of AMP-*g*-PAA in aqueous solution (pH = 4.8) as a function of ionic strength variation.

The zeta potential and particle size analysis of the copolymer AMP-*g*-PAA highlights the behavior in aqueous media as a function of pH (Figure 5) and ionic strength (Figure 6). Figure 5a shows that the copolymer AMP-*g*-PAA retains the same negative zeta potential throughout the entire investigated pH range as the PAA homopolymer, with the variation in values being similar for both; the negative charge seem to increase with the increase in solution pH. The AMP-*g*-PAA has a lower negative zeta potential as compared to PAA due to the contribution of the neutral AMP backbone to the overall copolymer structure.

The copolymer’s isoelectric point (zeta potential equal to zero) is located at about pH 3, and the same as observed for PAA, which indicates that the negative zeta potential values of the copolymer are correlated with the deprotonation of carboxyl groups from PAA grafted chains. Also, PAA shows high negative zeta potential values (−62 mV) at pH higher than 6, above its acidity constant (pKa ~ 4.5) [46], suggesting the maximum deprotonation of the carboxylic groups. The small changes in the zeta potential values encountered in the 6–9 pH range are related to electrostatic repulsions that lead to the rearrangement of polymeric chains in aqueous solution, thus exposing or hindering the completely ionized carboxyl groups. Similar to PAA, AMP-*g*-PAA shows a slight zeta potential value variation in the 6–10 pH domain that can also be ascribed to the full ionization of –COO^−^ groups.

In addition, the particle size distribution profiles (Figure 5b) and the scattered intensity (Figure 5c) of AMP-*g*-PAA self-assembled structures, following the pH variation, were measured by DLS. Since PAA is an anionic polyelectrolyte containing –COOH groups that are sensitive to pH, the conformational changes of AMP-*g*-PAA are also expected to vary depending on the solution pH, and thus, the sizes of the formed nanostructures in aqueous solution changed. Taking into account the partial ionization of the –COOH groups in the 3–5 pH range, AMP-*g*-PAA shows uneven particle size distribution, with a slight aggregation tendency as a consequence of low electrostatic repulsions among the carboxylate groups and the possibility of hydrogen-bond formation. The decrease in scattered intensity in the same pH range (3–5) indicates shrinking of the macromolecular chains in the aggregates and the formation of more compact structures with a polydispersity index (PDI) of about 0.27, possibly followed by partial disaggregation. At pH > 5, the increase in carboxyl groups deprotonation led to the increase in macromolecular aggregates size due to the electrostatic repulsions between similarly charged groups, resulting in the stretching of the copolymer molecular chains and an enhanced swelling capacity. By further increasing the pH from 8 to 10, a reorganization of the particle size distribution is observed, as evidenced by the broadening of the distribution peak in the 30–300 nm range and a PDI ranging in the domain 0.25–0.28, which is related to the macromolecular conformational changes occurring as a result of intensified electrostatic repulsions between the –COO^−^ functional groups. This may lead to further disaggregation of the formed aggregates. Therefore, the presence of particles under 100 nm can be ascribed to the formation of smaller polymer aggregates in alkaline medium.

The influence of the ionic strength on the AMP-*g*-PAA aqueous solution, as reflected in particle size distribution, was also investigated (Figure 6). The AMP-*g*-PAA chains exhibit an expanded conformation in aqueous media, assigned to the strong electrostatic repulsion among the negatively charged groups on the PAA grafted chains. By adding NaCl to the copolymer solution, the presence of counterions serves to effectively screen the electrostatic interactions, being responsible for the gradually collapsing of PAA chains grafted onto the AMP backbone (Figure 6a). This screening reduces both inter- and intrachain electrostatic repulsion, thereby promoting chain association and aggregation. The initial particle size distribution shows a dominant peak in the 100–900 nm range, which progressively shifts to lower sizes by raising the solution ionic strength up to 0.1 M, which remains almost constant for further increase up to 0.5 M (Figure 6c), suggesting chain contraction due to the decrease in electrostatic repulsions as a result of salt addition but also the stability of formed aggregates in the studied range of ionic strength, as is also supported by the PDI variation in the range 0.25–0.3. A secondary, low-intensity peak can be seen at higher sizes above 1000 nm, which can be attributed to higher aggregates that occur as a result of several factors, such as electrostatic repulsions between –COO^−^ groups, hydrogen bonds that can form between –COOH/–COOH and –COOH/–COO^−^ groups, or interactions between Na^+^ ions and –COO^−^ groups. However, the absence of a pronounced shift toward larger particle sizes confirms that no severe aggregation occurs.

Additionally, according to DLS measurements, a sharp decrease in the hydrodynamic diameter (Figure 6b) and an increase in PDI values from 0.255 to 0.266 were observed upon the first salt addition (0.05 M), which afterward remained relatively stable at approximately 150–200 nm regardless of further increases in salt concentration. This behavior can be associated with the deprotonation of –COOH groups in aqueous medium (pH = 4.8), which can further interact electrostatically with Na^+^ ions. As such, the majority of deprotonated groups interacted with Na^+^ ions, favoring aggregate formation as a result of reduced electrostatic repulsions and enabling hydrogen bonds and hydrophobic interactions. Consequently, the copolymer chains undergo slight contraction, leading to a compact structure. Importantly, regardless of the high NaCl concentration tested, there is no large increase in the particle size of AMP-*g*-PAA, indicating that the copolymer remains appropriately stabilized, and no major aggregation occurs.

### 3.3. AMP-g-PAA/AuNPs Composites Formation

The obtained AMP-*g*-PAA copolymer was further investigated regarding its ability to facilitate the in situ formation of AuNPs leading to colloidal organic/inorganic composites. The large amount of hydroxyl and carboxylic functional groups of the copolymer is expected to present a great potential for interaction with Au^3+^ ions, allowing their reduction to Au^0^ form in the polymer matrix and further stabilizing the formed AuNPs [57].

To find the optimum reaction parameters, the weight ratios HAuCl_4_/AMP-*g*-PAA of 0.28 and 0.36 were tested, tracking the impact of incubation duration at 60 °C (from 1 to 6 h), continued by storage for 10 days at room temperature, on achieving AuNPs embedded in the formed composite nanostructures (Figure 6 and Figures S4–S7 in Supplementary Information).

**Figure 7:**
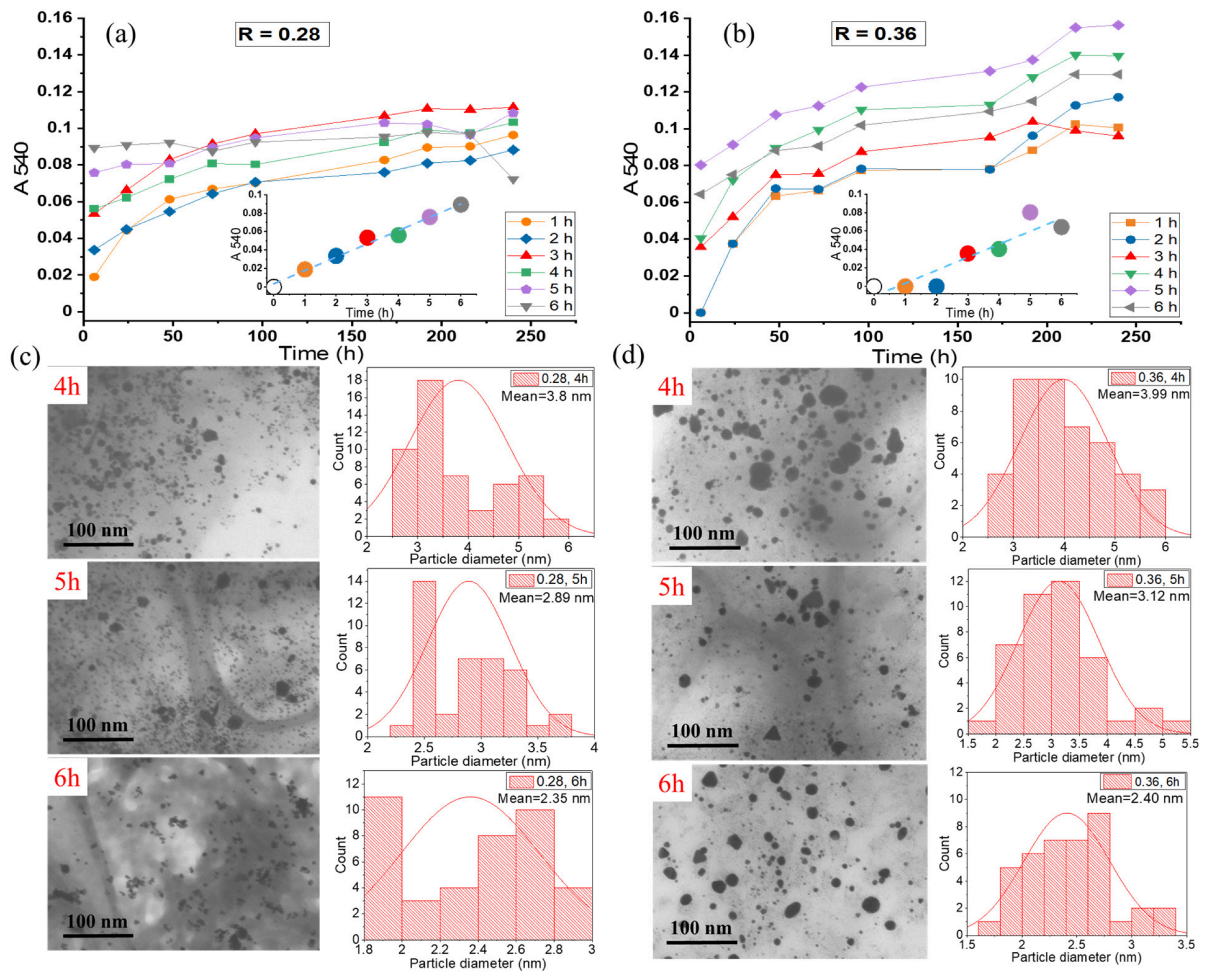
Formation of AuNPs at the weight ratio of HAuCl_4_/AMP-*g*-PAA of 0.28 (**a**,**c**) and 0.36 (**b**,**d**), followed by UV–vis spectroscopy at 540 nm at different incubation durations at 60 °C (insets in **a**,**b**), followed by 10 days storage at room temperature (**a**,**b**) and by STEM images and the corresponding histograms after 4 and up to 6 h at 60 °C and 10 days at room temperature (**c**,**d**).

**Scheme 2:**
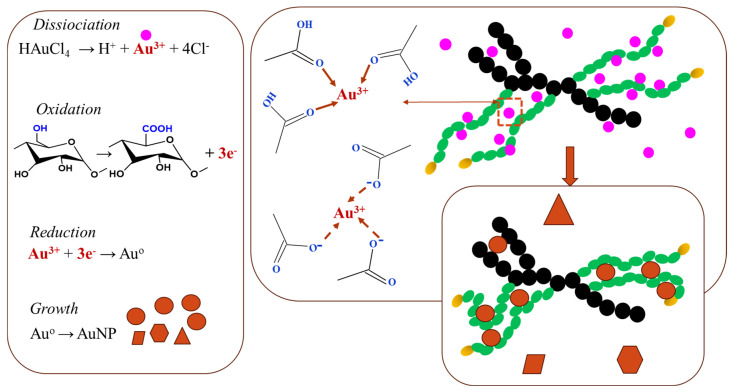
Proposed mechanism for AuNPs formation within the AMP-*g*-PAA copolymer matrix.

UV–vis spectra (Figure 7a,b, Figures S4 and S6) indicated the formation of AuNPs, showing an increase in the absorbance at 540 nm with time in a characteristic LSPR band of AuNP. At low incubation time (1–2 h, Figure 6a,b), the LSPR band was found to be fairly weak and broad (R = 0.28) or even absent (R = 0.36), suggesting that at this early stage, few Au^3+^ ions were reduced to metallic Au and formed very small amounts of AuNPs with a large variation in size (as observed by DLS size distribution curves; Figures S5 and S7). Increasing the reaction duration at 3–6 h, the LSPR signal increased in a more pronounced manner, indicating the accelerated growth rate of AuNP and also reflecting a more uniform size of the AuNP population due to the coordination of Au species with AMP-*g*-PAA functional groups. Also, the broad size distribution was found for samples incubated at 60 °C for 1–5 h irrespective of the duration of the storage at room temperature, whereas two populations are clearly evident for the samples, which were incubated for 6 h at 60 °C, suggesting that this was the best incubation time. Also, it may be assumed that the population with smaller-sized nanoparticles (5–20 nm) can be assigned to dispersed AuNP (sizes similar to that measured in the STEM images in Figure 7c,d), whereas the population with larger sizes (30–200 nm) represents the formed nanocomposites of AMP-*g*-PAA with AuNPs retained by different mechanisms (as suggested in Scheme 2). Also, it was found that for an incubation time of 1–3 h, some aggregates of AMP-*g*-PAA/AuNPs were formed but together with dispersed AuNPs with different sizes, as supported by the UV–vis and DLS measurements (Figures S4–S7), whereas increasing the incubation time to 4–6 h leads to the most uniformly distributed and well-defined and smaller AuNPs with less aggregation tendency, as are also evidenced in the STEM images in Figure 7c,d. At the longest reaction time (6 h, R = 0.36), all the observed AuNPs have spherical shape, their size decreased, and their dispersibility in the system increased. The decrease in the particle size for the longest reaction time is attributed to possible post-growth restructuring process in the system, which may promote partial dissolution and reprecipitation as well as fragmentation of loosely bound aggregates by Ostwald ripening and interparticle rearrangement [58]. As a result, even the average particle size seems to decrease at extended reaction times compared to the peak growth stage; actually, the system is reaching favorable stability due to its tendency to minimize the surface energy of formed particles.

Scheme 2 depicts a proposed mechanism for the formation of AuNPs in the presence of AMP-*g*-PAA. The ability of chloroauric acid (HAuCl_4_) to dissociate in aqueous solution into tetrachloroaurate ions (AuCl_4_^−^) and hydrogen ion is well-known. Thus, in the proposed mechanism, it is expected that the hydroxyl groups of AMP would participate in an oxidation process that leads to the formation of carboxyl groups, whereas the Au^3+^ ions are reduced to metallic gold (Au^0^) via the electron transfer mechanism of Au^3+^ + 3e^−^→Au^0^ in the first step. In the next step, the resulting Au^0^ species nucleate, forming the first cluster, which further aggregates in AuNPs close to the AMP-*g*-PAA chains, being stabilized by the secondary hydroxyl and carboxylic groups of the copolymer. This method of synthesizing well-dispersed and stable AuNPs embedded within the AMP-*g*-PAA matrix does not require any additional reducing/capping agents and is a simple and environmentally friendly approach to synthesizing AuNPs.

**Figure 8:**
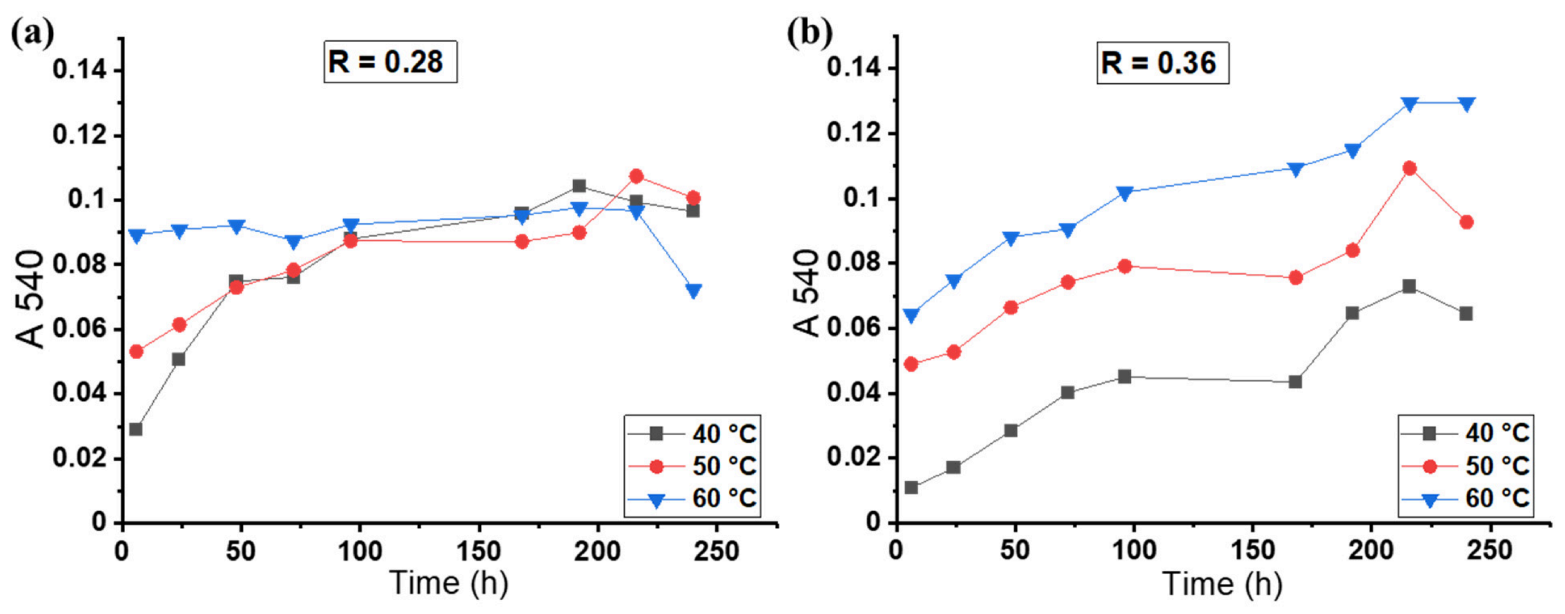
Formation of AuNPs at HAuCl_4_/AMP-*g*-PAA of 0.28 (**a**) and 0.36 (**b**) and at different incubation temperatures (40–60 °C) followed by UV–vis spectroscopy at 540 nm recorded for 10 days storage at 25 °C. (The results at 60 °C are introduced for comparison).

Taking into account the above results, the incubation time of 6 h was selected as the best synthesis condition, and in the following, the influence of the weight ratio HAuCl_4_/AMP-*g*-PAA was further investigated along with the influence of the incubation temperature (40–60 °C) (Figure 8 and Figures S4–S12 in Supplementary Information).

The UV–vis spectra (Figures S8–S10) show the development of the characteristic LSPR band of AuNPs at 540 nm, confirming their formation at all investigated incubation temperatures (Figure 8). The UV–vis spectra show a gradual increase in LSPR absorbance with an increasing amount of HAuCl_4_, namely by increasing the inorganic/polymer ratio from 0.28 to 0.36, and it depends on the incubation temperature. Thus, at R = 0.28, weak LSPR bands are observed regardless of temperature for storage times higher than 2 days, indicating the maximum reducing capacity of the AMP-*g*-PAA copolymer in the working conditions (Figure 8a). Also, the nearly constant LSPR absorbance value suggests good particle stabilization when the ratio is 0.28 (Figure 8a). At R = 0.36, narrow and well-defined LSPR bands are observed, suggesting uniform nanoparticle formation and effective steric stabilization by AMP-*g*-PAA, clearly controlled by the incubation temperature (Figure 8b). At 60 °C, the highest absorbance values indicate rapid reduction and higher AuNPs yield. This behavior indicates the temperature-sensitive growth dynamics of AuNPs. This confirms the importance of an optimal molar ratio for controlled nanoparticle formation. At lower inorganic/polymer ratio and lower temperature, peak broadening is observed, implying particle growth or partial aggregation (Figures S8–S10).

**Figure 9:**
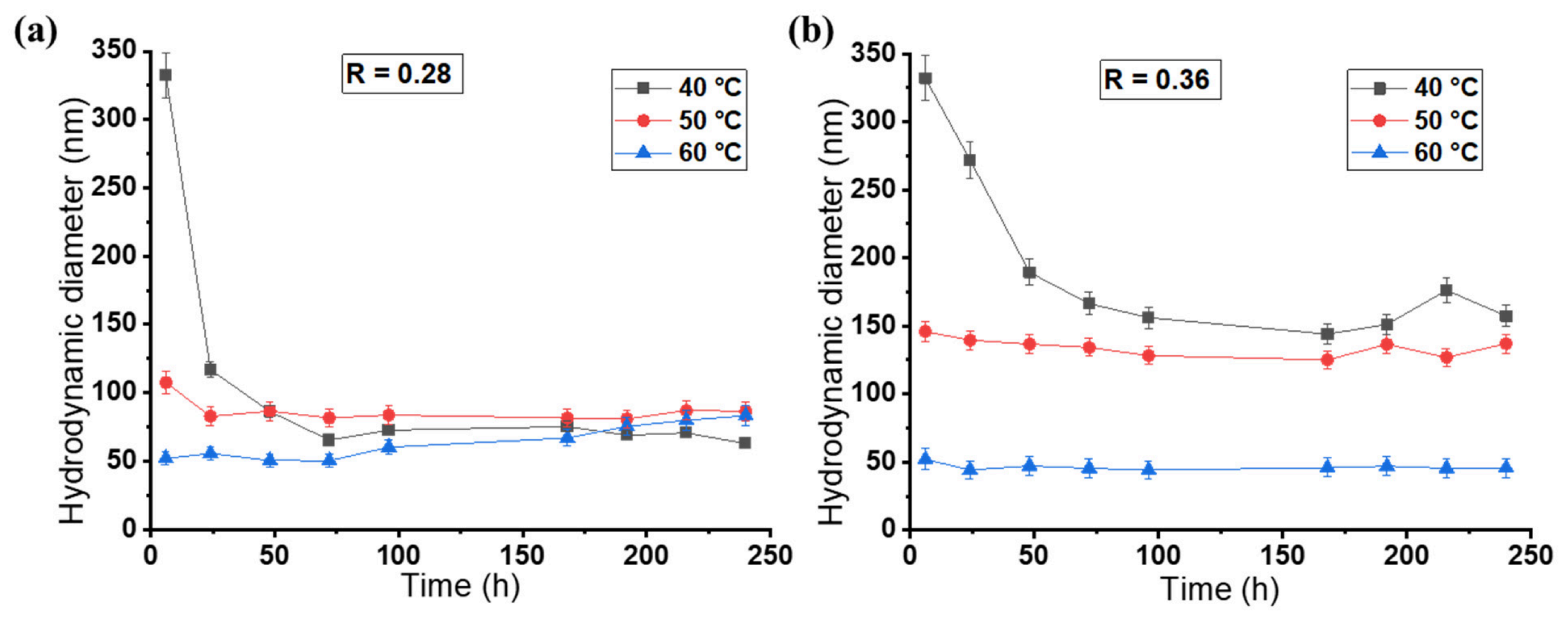
Hydrodynamic diameter of formed composites at the weight ratio of HAuCl_4_/AMP-*g*-PAA of 0.28 (**a**) and 0.36 (**b**) at different incubation temperatures (40–60 °C) for 6 h, followed by DLS for 10 days storage at 25 °C.

DLS results indicate that the development of AMP-*g*-PAA/AuNPs composites is very sensitive to both ratios between initial components and the incubation temperature. Figure 9 illustrates the variation of the hydrodynamic diameter (obtained from the size distribution intensity curves in Figures S5, S7, S11 and S12) of the AMP-*g*-PAA/AuNPs composites synthesized at 40 °C, 50 °C, and 60 °C using the [HAuCl_4_]/[AMP-*g*-PAA] ratio of 0.28 and 0.36.

The results presented in Figure 9a correspond to the variation in hydrodynamic diameter of AMP*-g*-PAA/AuNPs nanocomposites obtained with a ratio of 0.28 at 40 °C, 50 °C, and 60 °C after 6 h incubation time. At this ratio, by changing the temperature from 40 °C to 60 °C, an initial reduction in the hydrodynamic diameter (and a decrease in PDI to 0.286) can be seen due to increased nucleation rates, where the particle hydrodynamic diameter values are almost constant during the 1–10 days storage and are slightly influenced by the incubation temperature. This observation demonstrates that the gold ions are completely reduced and stabilized by the polymer matrix. Increasing the inorganic/polymer ratio to 0.36 (Figure 9b) and increasing the incubation temperature from 40 to 60 °C decreases the hydrodynamic diameter (and decreases PDI to 0.276), indicating that the reduction kinetics is favored by temperature and that the nucleation process occurs more rapidly. AuNPs formed at 40 °C display the higher average diameters, whereas increasing the temperature to 60 °C causes a reduction in size and a more rapid nucleation with composite particles stabilization during the entire storage period of ten days.

From all the information provided above, it can be concluded that the temperature consistently influenced the composite’s hydrodynamic diameter, with a higher thermal energy promoting larger chain movement and enhanced intermolecular forces. Accordingly, we can conclude that the best conditions for producing AuNPs are a ratio of 0.36 of inorganic/polymer components and a 6 h incubation time at 60 °C, producing well-defined, stable AMP-*g*-PAA/AuNPs for future applications.

As can be seen in Table 1, most of the synthesis methods involve higher temperature or special reaction conditions for the formation and stabilization of AuNPs (i.e., acetic acid, irradiation, etc.). On the contrary, in our study, the reaction temperature was 60 °C, and the AuNPs were obtained in the presence of the graft copolymer without additional reduction agents. Moreover, we have to mention that we did not find in the literature tests on AMP grafted copolymers used in AuNPs synthesis or in the conditions applied in this study.

**Figure 10:**
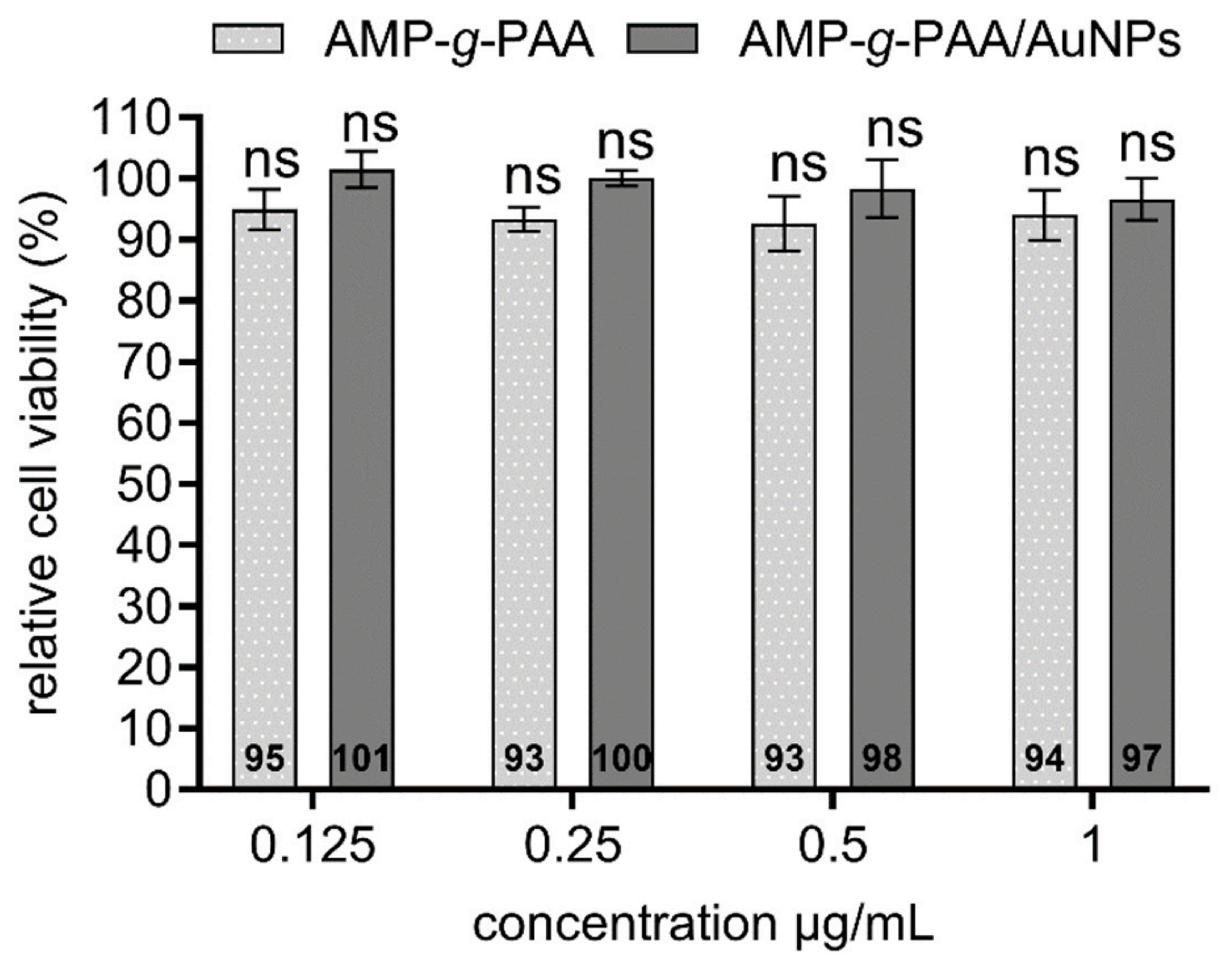
Cytotoxicity assessment of AMP-*g*-PAA and AMP-*g*-PAA/AuNPs in human dermal fibroblasts (HDF) after 48 h exposure. Relative cell viability (%) of HDF cells treated with increasing concentrations (0.125, 0.25, 0.5, and 1 µg/mL) of AMP-*g*-PAA (dotted light grey bars) and AMP-*g*-PAA/AuNPs (solid dark grey bars), as determined by the CellTiter-Glo^®^ Luminescent Cell Viability Assay. Cell viability was normalized to the untreated negative control and measured by ATP-dependent bioluminescence. Numerical values inside each bar indicate mean relative cell viability (%). Data are expressed as mean ± standard deviation from three independent experiments performed in duplicate. Statistical significance was assessed by two-way ANOVA, followed by Dunnett’s multiple comparisons test against the untreated control. ns = not significant (*p* > 0.05).

Taking into consideration the above observations, this particular composite together with the AMP-*g*-PAA copolymer were selected for cytotoxicity tests (Figure 10), with different quantities from each sample being added over the HDF culture media in order to evaluate the cellular viability at different concentrations.

As seen in Figure 9, the relative cell viability after 48 h was maintained over 90% for both investigated samples and for all the tested concentrations (0.125–1 µg·mL^−1^), which, according to the literature, are considered extremely high, indicating that the samples are biocompatible. Even better, the cells with AMP-*g*-PAA/AuNPs showed even higher relative cell viability values (96%) as compared to the corresponding concentrations of the copolymer (94%), confirming the absence of significant dose-dependent cytotoxicity. The high biocompatibility of AMP-*g*-PAA is consistent with the generally recognized low-toxicity profile of polysaccharide-based polymers, particularly those derived from natural biopolymers such as amylopectin. Notably, the incorporation of AuNPs synthesized in situ within the polymer matrix did not introduce additional cytotoxicity compared to the parent copolymer alone. Overall, these results support the biocompatibility of both formulations in a dermal cell model and provide a promising safety baseline for further investigation in applications such as drug delivery, wound healing, or other biomedical applications.

## 4. Conclusions

In this study, we successfully synthesized and characterized an AMP-*g*-PAA graft copolymer by RAFT polymerization using a “grafting to” strategy. Spectroscopic analyses (FTIR and 1D and 2D NMR) confirmed successful covalent attachment of PAA side chains, and thermal studies showed improved stability compared to the precursor polymers. The copolymer demonstrated typical polyelectrolyte behavior vs. pH and ionic salt modification and acted as an efficient macromolecular platform to realize the green synthesis of gold nanoparticles without the use of any extra reducing agents. By systematically studying the relationship between temperature and the ratio between initial components, it was found that the higher temperature increases the nucleation of AuNPs, decreases the aggregation tendency, and provides more uniform composite nanostructures. The most stable colloidal nanocomposites were obtained at R = 0.36 and a temperature of 60 °C for a 6 h reaction time, as shown by UV–vis, DLS, and STEM analysis. Selected AMP-*g*-PAA/AuNPs nanocomposites were evaluated in terms of cytotoxicity, revealing a relative cell viability above 90% for all tested concentrations. Overall, this investigation illustrates that the AMP-*g*-PAA copolymer offers the potential to act as an environmentally friendly and effective component for the controlled synthesis of AuNPs, recommending the formed composites for biomedical applications.

## Figures and Tables

**Table 1:** Literature survey on polysaccharide-based systems for green AuNP synthesis.

Polysaccharide	Reaction Temp (°C)	Reaction Special Conditions	Reaction Time (min)	Ref.
starch	70	stirring 350 rpm, pH = 12	1	[59]
alginate	70	glucose	30	[60]
alginate	microwave	ethylene glycol	1–3	[61]
chitosan	irradiation	acetic acid	-	[62]
chitosan	50–90	pH 2–6	20–100	[63]
chondroitin sulfate	room temperature	sodium borohydride	20	[64]
chondroitin sulfate A	80	-	300	[65]
laminarin	80	-	20	[66]
carboxymethyl cellulose	85	stirring	240	[67]
chitin	25	p-mercaptobenzoic acid, stirring 500 rpm, CO bubbling	4320 min (72h)	[68]
this study	40–60	-	60–360	

## Data Availability

The original contributions presented in this study are included in the article or Supplementary Materials. Further inquiries can be directed to the corresponding author.

## Supplementary Materials

The following supporting information can be downloaded at: https://www.mdpi.com/article/10.3390/polym18131636/s1. Figure S1: Size-exclusion chromatography (SEC) results of PAA; Figure S2: ATR-FTIR spectra of AMP, AMP/PAA physical mixture, and AMP-***g***-PAA copolymer; Figure S3: DEPT and COSY NMR spectra of AMP and AMP-*g*-PAA copolymer; Figure S4: UV–vis spectra of colloidal nanocomposites prepared with the weight ratio HAuCl_4_/AMP-*g*-PAA = 0.28 at different reaction duration at 60 °C (1–6 h) and for 10 days storage at room temperature; Figure S5: Size distribution of colloidal nanocomposites prepared with the weight ratio HAuCl_4_/AMP-*g*-PAA = 0.28 at different reaction durations at 60 °C (1–6 h) and for 10 days storage at room temperature; Figure S6: UV–vis spectra of colloidal nanocomposites prepared with the weight ratio HAuCl_4_/AMP-*g*-PAA = 0.36 at different reaction durations at 60 °C (1–6 h) and for 10 days storage at room temperature; Figure S7: Size distribution of colloidal nanocomposites prepared with the weight ratio HAuCl_4_/AMP-*g*-PAA = 0.36 at different reaction durations at 60 °C (1–6 h) and for 10 days storage at room temperature; Figure S8: UV–vis spectra of colloidal nanocomposites prepared with the weight ratios HAuCl_4_/AMP-*g*-PAA of 0.28 and 0.36 incubated at 40 °C for 6 h and for 10 days storage at room temperature; Figure S9: UV–vis spectra of colloidal nanocomposites prepared with the weight ratios HAuCl_4_/AMP-*g*-PAA of 0.28 and 0.36 incubated at 50 °C for 6 h and for 10 days storage at room temperature; Figure S10: UV–vis spectra of colloidal nanocomposites prepared with the weight ratios HAuCl_4_/AMP-*g*-PAA of 0.28 and 0.36 incubated at 60 °C for 6 h and for 10 days storage at room temperature; Figure S11: Size distribution of colloidal nanocomposites prepared with the weight ratios HAuCl_4_/AMP-*g*-PAA of 0.28 and 0.36 incubated at 40 °C for 6 h and for 10 days storage at room temperature; Figure S12: Size distribution of colloidal nanocomposites prepared with the weight ratios HAuCl_4_/AMP-*g*-PAA of 0.28 and 0.36 incubated at 50 °C for 6 h and for 10 days storage at room temperature.
