# Supplementary material for: Amylopectin-g-Poly(Acrylic Acid): Synthesis and Application as Reduction Agent for In Situ Formation of Gold Nanoparticles
Source: Polymers (Basel). 2026 Jul 1;18(13):1636. doi: 10.3390/polym18131636 (PMC13364450; doi:10.3390/polym18131636)
Supplement: Supplementary file 1 [file polymers-18-01636-s001.zip › polymers-4372230-supplementary.pdf]

Supplementary information’s

Amylopectin-g-poly(acrylic acid). Synthesis and application as reduction agent for in situ formation of gold nanoparticles

Melinda-Maria Bazarghideanu, Marius-Mihai Zaharia, Florin Bucatariu, Ana-Lavinia Vasiliu, Marcela Mihai*, Stergios Pispas*

Petru Poni Institute of Macromolecular Chemistry, 41A Grigore Ghica Voda Alley, 700487 Iasi, Romania; melinda.bazarghideanu@icmpp.ro (M.-M.B); zaharia.marius@icmpp.ro (M.-M.Z.); fbucatariu@icmpp.ro (F.B.); vasiliu.lavinia@icmpp.ro (A.-L.V)

* Correspondence: marcela.mihai@icmpp.ro (M.M.); pispas@eie.gr (S.P.)

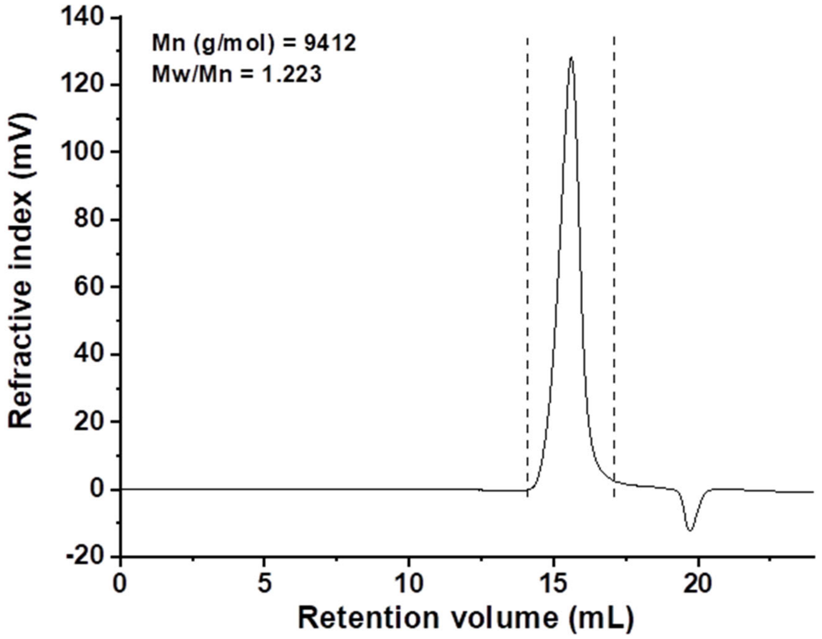

Figure S1. Size-exclusion chromatography (SEC) results of PAA.

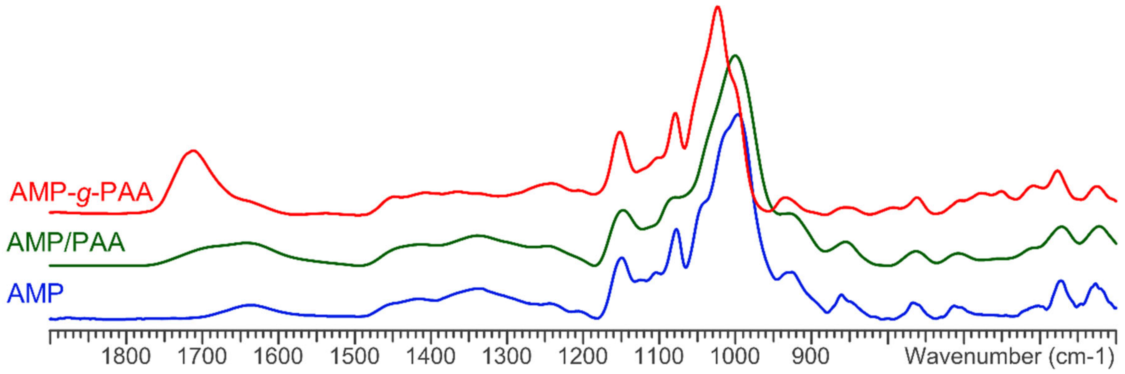

Figure S2. ATR-FTIR spectra of AMP, AMP/PAA physical mixture and AMP-*g*-PAA copolymer.

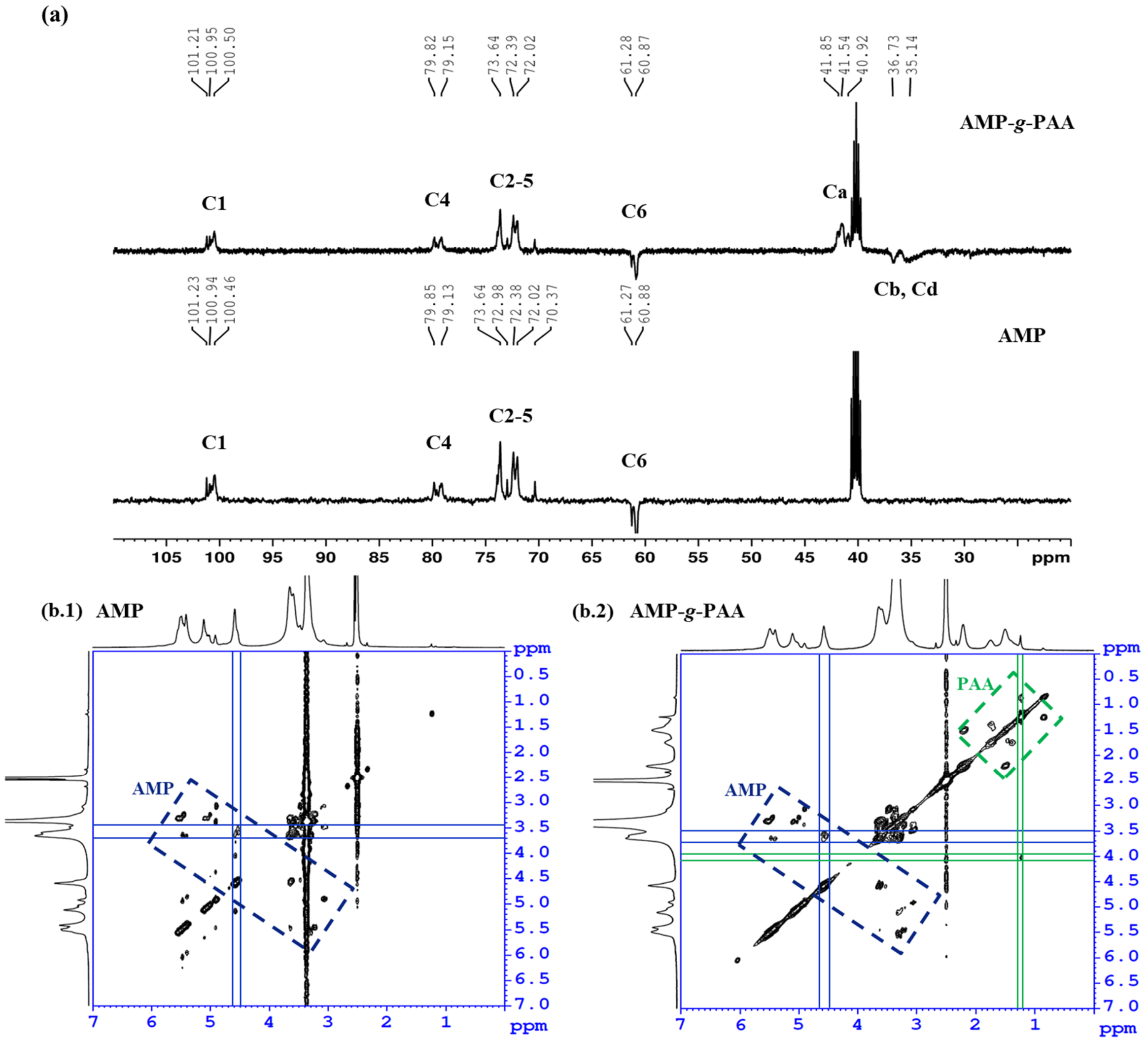

Figure S3. DEPT and COSY NMR spectra of AMP and AMP-*g*-PAA copolymer.

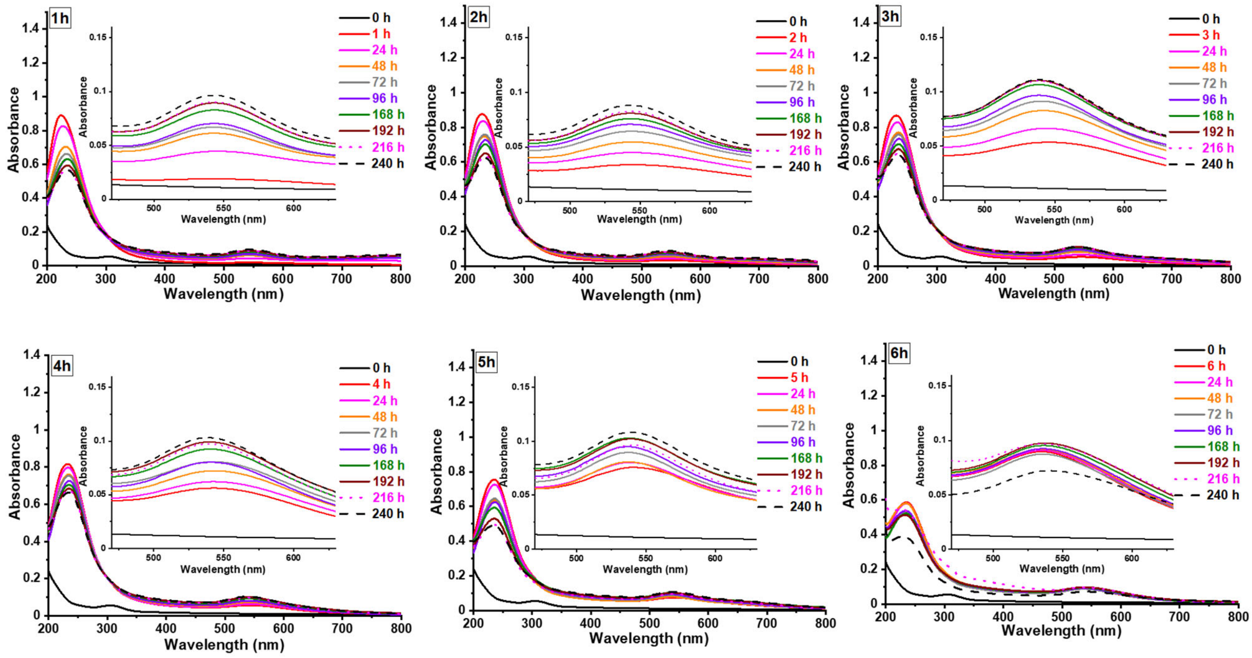

Figure S4. UV-Vis spectra of colloidal nanocomposites prepared with the weight ratio $HAuCl_4$/AMP-*g*-PAA = 0.28 at different reaction duration at 60 °C (1-6 h) and for 10 days storage at room temperature.

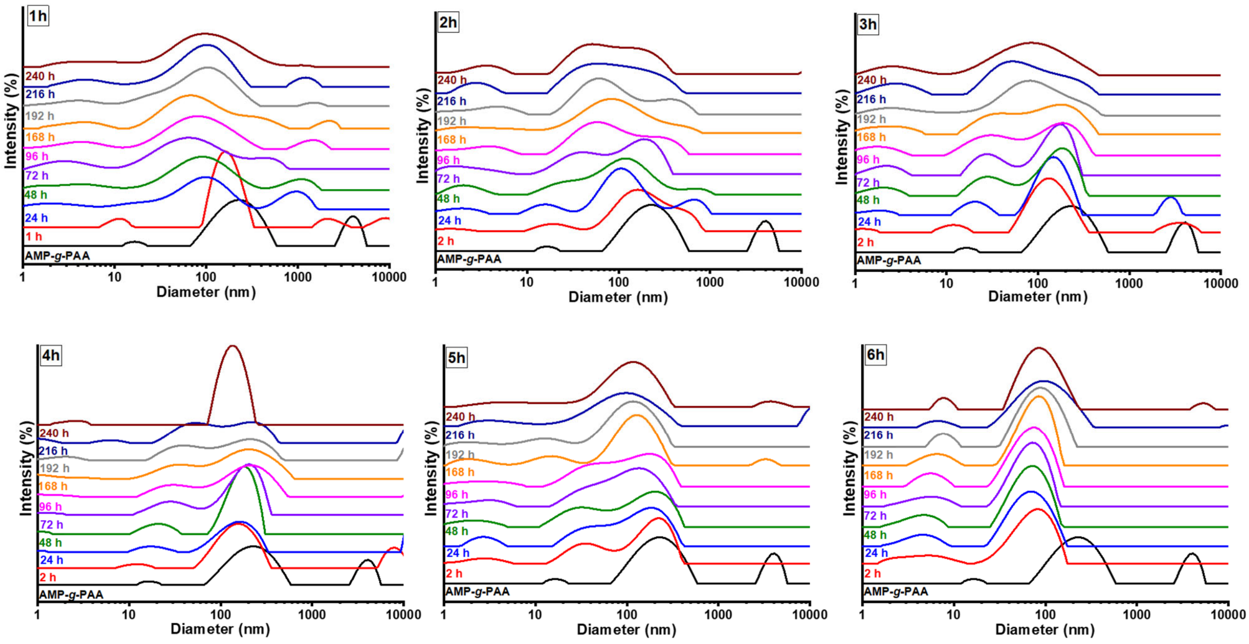

Figure S5. Size distribution of colloidal nanocomposites prepared with the weight ratio $HAuCl_4$/AMP-*g*-PAA = 0.28 at different reaction duration at 60 °C (1-6 h) and for 10 days storage at room temperature.

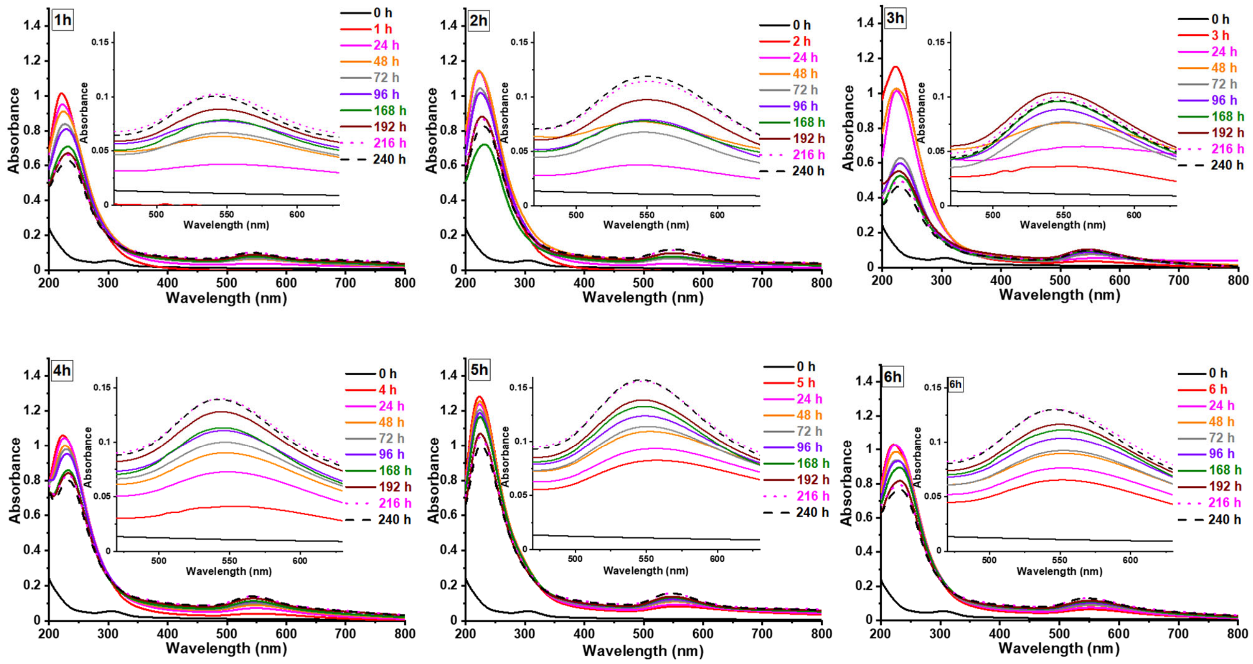

Figure S6. UV-Vis spectra of colloidal nanocomposites prepared with the weight ratio $HAuCl_4$/AMP-*g*-PAA = 0.36 at different reaction duration at 60 ^{o}C (1-6h) and for 10 days storage at room temperature.

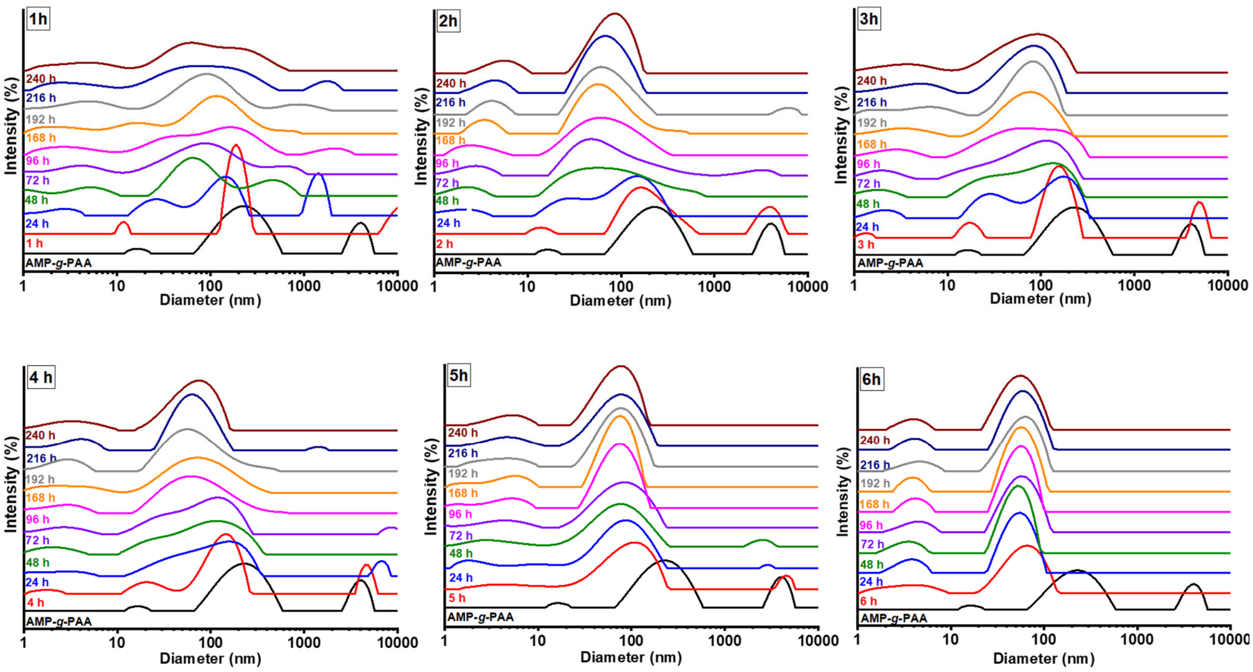

Figure S7. Size distribution of colloidal nanocomposites prepared with the weight ratio $HAuCl_4$/AMP-*g*-PAA = 0.36 at different reaction duration at 60 °C (1-6 h) and for 10 days storage at room temperature.

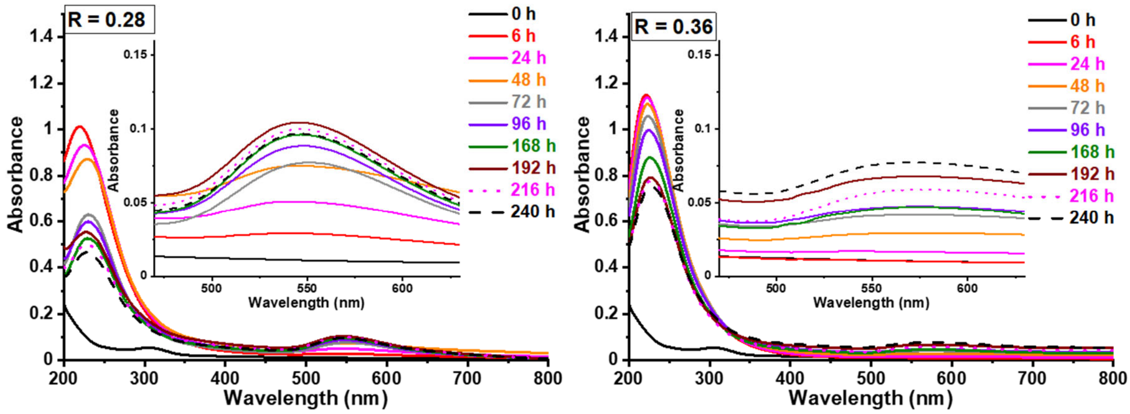

Figure S8. UV-Vis spectra of colloidal nanocomposites prepared with the weight ratios $HAuCl_4$/AMP-*g*-PAA of 0.28 and 0.36 incubated at 40°C for 6h and for 10 days storage at room temperature.

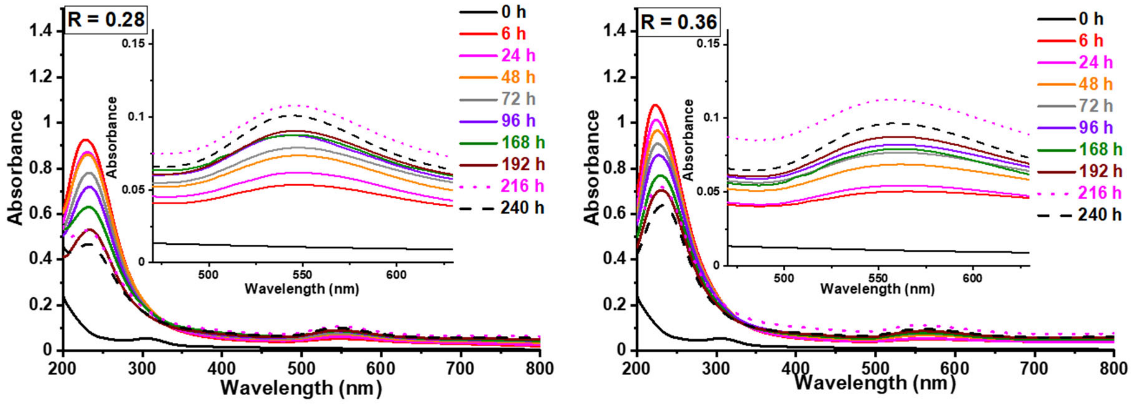

Figure S9. UV-Vis spectra of colloidal nanocomposites prepared with the weight ratios $HAuCl_4$/AMP-*g*-PAA of 0.28 and 0.36 incubated at 50°C for 6h and for 10 days storage at room temperature.

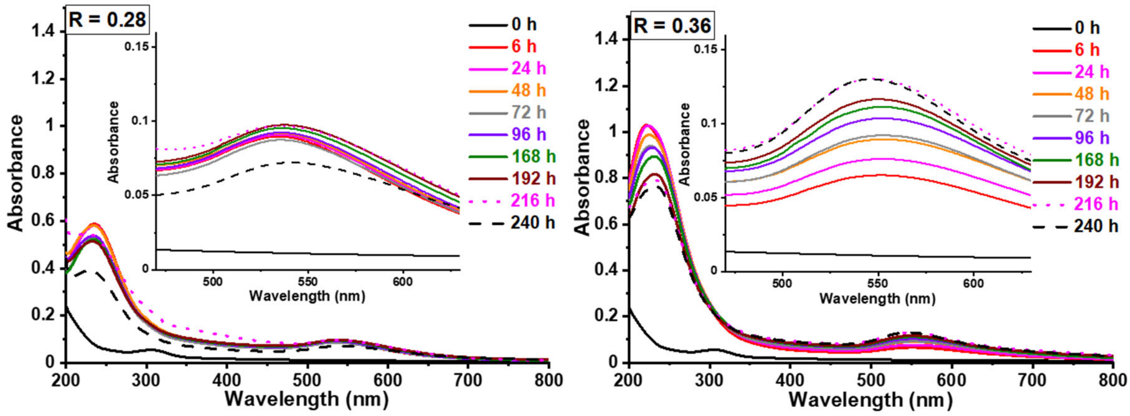

Figure S10. UV-Vis spectra of colloidal nanocomposites prepared with the weight ratios $HAuCl_4$/AMP-*g*-PAA of 0.28 and 0.36 incubated at 60°C for 6h and for 10 days storage at room temperature.

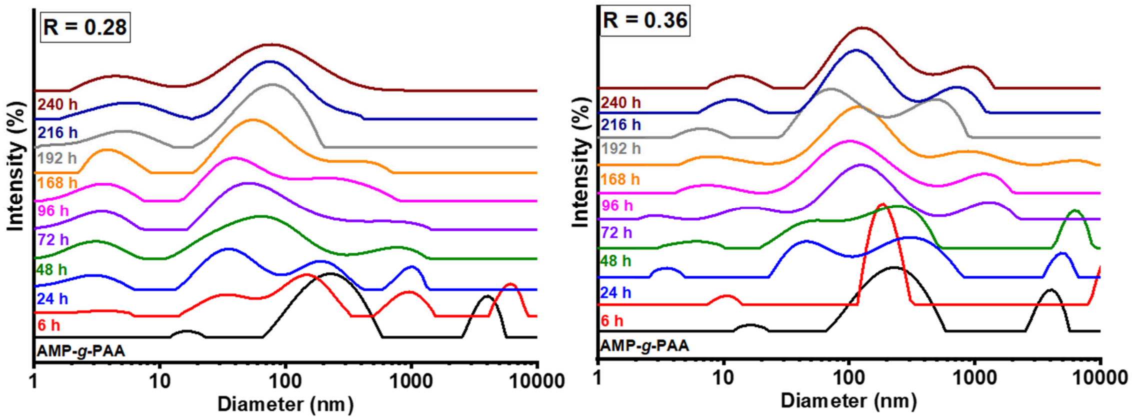

Figure S11. Size distribution of colloidal nanocomposites prepared with the weight ratios $HAuCl_4$/AMP-*g*-PAA of 0.28 and 0.36 incubated at 40°C for 6h and for 10 days storage at room temperature.

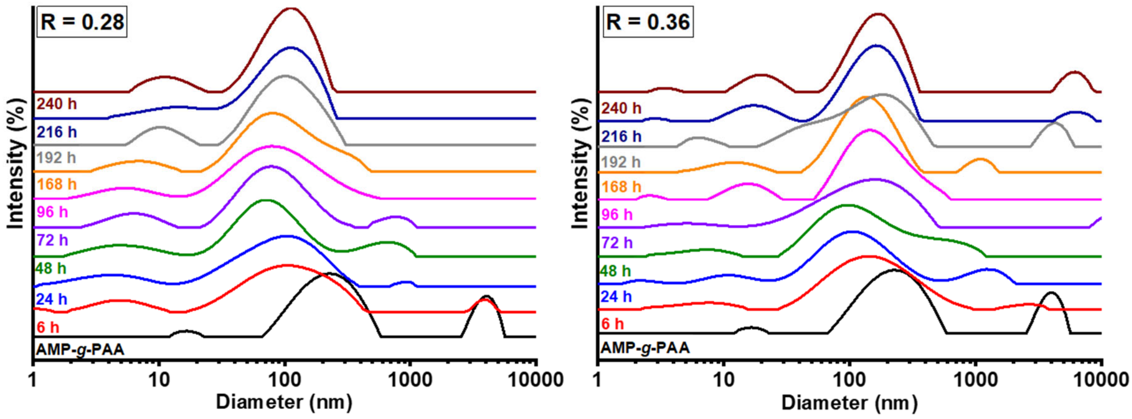

Figure S12. Size distribution of colloidal nanocomposites prepared with the weight ratios $HAuCl_4$/AMP-*g*-PAA of 0.28 and 0.36 incubated at 50°C for 6h and for 10 days storage at room temperature.
